# Impact of Patient Portals on Asthma and Chronic Obstructive Pulmonary Disease–Related Quality-of-Care Outcomes: Systematic Review

**DOI:** 10.2196/83483

**Published:** 2026-08-24

**Authors:** Sukriti Kc, Nicholas S Hopkinson, Ana Luisa Neves, Azeem Majeed, Anthony A Laverty

**Affiliations:** 1 Department of Primary Care and Public Health, Imperial College London, 90 Wood Lane, London, W12 0BZ, United Kingdom, 020 7589 5111; 2 National Heart and Lung Institute, Imperial College London, London, United Kingdom; 3 Global Digital Health Unit, Imperial College London, London, United Kingdom

**Keywords:** patient portal, asthma, COPD, electronic health record, digital health

## Abstract

**Background:**

Patient portals are increasingly used to support self-management and facilitate care coordination in people with chronic diseases. Although some asthma quality-of-care gains and modest chronic obstructive pulmonary disease (COPD) improvements have been reported in wider digital health studies, a comprehensive review of patient portal-specific evidence is lacking.

**Objective:**

This study aimed to systematically synthesize evidence on the impacts of patient portals on adult asthma and COPD outcomes across 6 quality-of-care domains (effectiveness, patient-centeredness, equity, efficiency, safety, and timeliness).

**Methods:**

We searched 5 databases (MEDLINE/PubMed, Embase, PsycINFO, CINAHL, and Google Scholar) for quantitative evaluations published up to March 2026 (CRD42022316044). The Newcastle-Ottawa Scale and Cochrane Risk of Bias-2 tool were used for quality assessments, and certainty of evidence was evaluated using the GRADE (Grading of Recommendations Assessment, Development, and Evaluation) approach. Findings were narratively synthesized using the SWiM (Synthesis Without Meta-analysis) framework.

**Results:**

We identified 4038 records, and 16 papers published between 2007 and 2024 met the inclusion criteria (asthma=13, COPD=2, both=1). Three were randomized controlled trials, all rated high risk of bias. Of the other studies, 5 had low risk, 6 had moderate risk, and 2 had high risk of bias. Effectiveness and patient-centeredness were most frequently reported, with 12 papers each. Eight papers were on equity, 5 each on efficiency and safety, and 4 on timeliness. In asthma, modest disease control improvements were reported in 27.8% (42/151) of participants using patient portals with home visit support versus portal use alone (35/150, 23.3%). In COPD, one large, interrupted time series study (N=909,724) reported post-implementation reductions in hospitalizations (slope change from 1.33 to −4.38 per 10,000 patients per month; *P*<.001) but no mortality benefits. Short-term improvements in asthma-related quality of life were observed among patient portal users (mean Mini Asthma Quality of Life Questionnaire score change of 0.67; 95% CI 0.36-0.98) but with no sustained effects. In terms of equity, several digital access barriers were noted. Assessments of efficiency outcomes were largely subjective, and safety events were weakly described. Across all outcome domains, GRADE assessment indicated low to very low evidence certainty.

**Conclusions:**

This review found low to very low certainty of evidence on the impacts of patient portals on quality of care for asthma and COPD. While modest disease control improvements and short-term quality-of-life gains were noted in asthma, and reduced hospitalizations in COPD were observed, the evidence base remains limited and methodologically weak. This review cautions against a reliance on patient portals for clinical management of asthma and COPD and highlights that more methodologically robust primary studies are needed to identify how portal functions may yield clinically meaningful benefits for patients with chronic respiratory disease.

## Introduction

Chronic respiratory diseases, including asthma and chronic obstructive pulmonary disease (COPD), affect approximately 450 million people worldwide [1]. Asthma prevalence in the United Kingdom ranks among the highest globally, with up to 8 million people living with asthma, and it causes 3 deaths each day [2,3]. Likewise, nearly 3 million people in the United Kingdom live with COPD, and it is one of the leading causes of emergency hospitalizations [4]. Together, these conditions contribute to a significant economic burden on the National Health Service (NHS), with an approximate annual cost of £3 billion (£1=US $1.33 as of July 27, 2026) for asthma and a further £1.9 billion for COPD [5]. Despite the scale of this burden, the quality of respiratory care in the United Kingdom remains suboptimal [6]. A recent national audit highlighted persistent shortfalls in respiratory care across England and Wales, with notable delays in timely care and missed opportunities for early intervention [6]. These gaps underscore the need for approaches that extend beyond episodic clinical encounters and support ongoing disease management.

With the advent of electronic health records (EHRs), digital services such as patient portals are increasingly recognized as tools that support patient engagement in chronic disease care [7]. As services that are linked to provider EHRs, patient portals typically provide access to personal health information, test results, and offer patient-provider communication options, which enable patients to more actively participate in their care processes [7]. These functions are particularly relevant for asthma and COPD, where symptom variability and frequent treatment adjustments are common, and effective management for both conditions relies on sustained patient engagement using shared decision-making strategies [8,9].

Evidence suggests that patient portals can support this role and offer several patient-specific benefits, such as timely access to health information, enhanced patient-provider communication, and improved care satisfaction [10,11]. Portal functionalities that allow remote assessment and real-time symptom checks may also support workflow efficiencies and contribute to more timely clinical review [12]. Among patients with chronic health conditions, patient portal use has been associated with improvements in selected patient engagement indicators, including disease knowledge, treatment adherence, and health care usage in some circumstances [13]. However, a well-documented limitation in the patient portal literature is the substantial variation in portal features, which are often tailored to disease-specific pathways, making it difficult to assume generalized benefits across conditions [14]. This highlights the need for a more focused evaluation in asthma and COPD to understand how patient portals influence the multidimensional needs of people living with these chronic respiratory diseases.

The Institute of Medicine provides one such framework for a structured evaluation of care quality across domains of effectiveness, safety, patient-centeredness, efficiency, timeliness, and equity impact [15-17]. Effectiveness relates to the delivery of evidence-based care and health outcome improvements, while safety refers to safeguards against errors and adverse events. Likewise, patient-centeredness is based on approaches that address individual needs, whereas efficiency relates to the avoidance of waste in the use of economic, human, and technical resources. This framework also considers timeliness, which refers to reductions in delays to care, and equity impacts, which relate to improvements in avoidable disparities in service provision [15].

Findings across care quality indicators for asthma and COPD remain fragmented and often do not separate patient portals from other technologies, or they are focused on a limited subset of outcomes. For example, a recent systematic review noted patient portal benefits related to some markers of effective disease management, such as medication adherence and reduced emergency visits, but this evidence was restricted to the pediatric population with asthma [18]. Another review identified some efficiency benefits, such as enhanced access for asthma care, but did not distinguish the specific contributions of patient portals from those of wider digital tools [19]. Similar improvements related to treatment adherence, unscheduled health care service use, and asthma control were also noted in a recent Cochrane review, but this study also did not isolate the effects of patient portals from other interventions [20]. A recent review further identified some patient portal-related improvements in exacerbation self-management and quality of life (QoL) in COPD, but the overall results remained inconclusive [21]. As no review currently provides a comprehensive overview of the care quality outcomes associated with patient portals in asthma and COPD, an updated evidence synthesis is required. Therefore, this systematic review aimed to provide up-to-date evidence on the impacts of patient portals on asthma and COPD, with a primary objective of evaluating effects across the 6 domains of quality of care.

## Methods

### Eligibility Criteria

We considered all primary research using quantitative study designs that included adults (aged 17 years and older) with asthma and/or COPD at any disease stage using patient portals. This age cutoff is in line with UK clinical practice guidelines for the management of respiratory conditions such as asthma in adults [22]. As there is no single definition of a patient portal, we used a pragmatic approach to define patient portals as provider EHR-linked tools that offer patients access to their personal health information and enable care coordination using functions such as record views, prescription requests, communication options, appointment booking, and so on [23-25]. To ensure the validity of the reported outcome measures, services that offer portal functions but lack health record integration (ie, stand-alone symptom trackers, medication reminder tools, symptom alert systems, etc) were excluded. Comparators included usual care or other forms of self-management, and studies were included if they reported on at least one of the 6 quality-of-care domains. Mixed methods studies were eligible for inclusion, but only quantitative outcome data were considered. The full eligibility criteria are detailed in Textbox 1.

Textbox 1. **Study inclusion and exclusion criteria used for the screening decision to identify all eligible studies.**

Inclusion criteria

- All quantitative study designs, including randomized controlled trials, cluster randomized trials, quasi-experimental, case-control, cohort studies, and so on.
- Studies including adults aged 17 years and older with a diagnosis of asthma or chronic obstructive pulmonary disease.
- Studies in English.
- All available studies published to date.

Exclusion criteria

- Studies that do not include primary empirical data.
- Services that are not linked to electronic health records, or those that provide non–electronic health record–based services such as appointment or instruction reminders, telemonitoring tools, and online educational platforms, as well as applications that rely on self-monitoring and self-recording of symptoms.
- Full text not available.

### Search Strategy

The review was conducted according to the PRISMA (Preferred Reporting Items for Systematic Reviews and Meta-Analyses) 2020 guidance and the SWiM (Synthesis Without Meta-analysis) reporting guideline [26,27]. The study protocol was registered with PROSPERO (International Prospective Register of Systematic Reviews) (CRD42022316044). The search strategy was developed and reported according to the PRISMA-S (Preferred Reporting Items for Systematic Reviews and Meta-Analyses literature search extension) guidelines [28]. Electronic searches of key databases such as MEDLINE(R), Embase, PsycINFO via OVID, CINAHL via EBSCOhost, and Google Scholar were conducted in January 2025 and updated in March 2026 using keywords and the MeSH terms from the National Library of Medicine. The full electronic search strategies for all databases, including complete Boolean search strings and limits used, are detailed in “Full Search Strategy for All Databases” in Multimedia Appendix 1 [29-44].

Bibliographies of included studies were also screened to identify additional papers not captured in the primary search. Attempts to find missing data and access to studies that potentially met the inclusion criteria but lacked full texts were made by contacting the corresponding authors and source journals. Analyses were conducted using the available data.

### Selection Process

Two reviewers (SK and AAL) independently conducted initial title and abstract screening, followed by full-text evaluation to determine study eligibility for inclusion. Covidence software was used for deduplication and screening, and all conflicts were resolved by discussion. Data extraction and quality assessment were conducted by 1 reviewer (SK) and checked by a second reviewer (AAL). Data were extracted using a standardized computer-based spreadsheet and included details of the study, population, intervention, comparator, and outcomes. Only quantitative data were extracted from mixed methods studies.

### Data Items and Synthesis

Outcomes were categorized using the Institute of Medicine (IOM) quality-of-care guide for evidence synthesis. The IOM framework offers a structured approach to understand the quality of healthcare services, and it has been previously used to assess digital health interventions [15,45]. Extracted data categorized according to the IOM framework included indicators of 1. Effectiveness (eg, disease and symptom management, medication use, activity limitation, lung function, etc), 2. Patient-centeredness (eg, indicators of self-management, knowledge, QoL, satisfaction, etc), 3. Equity impacts (eg, socioeconomic outcomes or other determinants of health), 4. Efficiency (eg, economic outputs, streamlined administrative tasks, etc), 5. Safety related to both patient health and technology (eg, adverse events, privacy, security, etc) and 6. Timeliness (eg, time to care, information exchange timeliness, etc).

This review followed the SWiM reporting guideline to ensure transparency in narrative synthesis [26]. Studies were grouped according to (1) disease type (asthma or COPD) and (2) the IOM quality-of-care domains (effectiveness, patient-centeredness, equity impacts, efficiency, safety, and timeliness). A narrative synthesis of results was performed as the studies had different designs, differing availability of and exposure to patient portal functionalities, and different outcomes assessed using a range of instruments. Additionally, there were few studies in each exposure-outcome pair and so, in line with other studies [18,46] and guidance [47], we did not consider that meta-analysis would be informative. Due to the limited number of studies per outcome, formal assessment of publication bias using funnel plots was also not performed and this was assessed narratively. Reported effect measures included mean differences, proportions, regression coefficients, and estimates of trends, depending on the study design and outcome type.

Outcomes were standardized by category with effects reported as positive, no effect, or negative within each domain in the visual representation using a harvest plot. A structured narrative synthesis was undertaken, supported by tabulation of study characteristics (Multimedia Appendix 2) and domain-level summaries (). In reporting, greater emphasis was placed on findings from studies with a lower risk of bias and larger sample sizes. Variability in results was explored in relation to differences in study design, population characteristics, portal functionalities, and follow-up duration.

### Risk-of-Bias Assessment

Quality assessment was using the original Newcastle-Ottawa Scale for cohort studies and an adapted version from a previous review for cross-sectional studies [48,49]. For randomized controlled trials (RCTs), the Cochrane Risk of Bias Tool 2.0 was used [50]. This was a deviation from the registered study protocol (PROSPERO:CRD42022316044). Although the protocol prespecified the use of the Joanna Briggs Institute Critical Appraisal Tool, the Newcastle-Ottawa Scale and the Cochrane Risk of Bias Tool 2.0 were ultimately used as they supported study design–specific assessment and domain-level evaluation, allowing for a more transparent and rigorous appraisal of bias. Quality assessments were carried out independently by 1 reviewer (SK) and verified by a second (AAL). The quality of evidence across each of the IOM domains was also assessed using the GRADE (Grading of Recommendations Assessment, Development and Evaluation) approach to support the strength of the recommendation [51,52]. Using GRADE, the narrative summary of effect for each outcome domain was assessed by considering the risk of bias across studies, their indirectness, imprecision, inconsistency, and publication bias [51,52].

## Results

### Study Selection

**Figure 1.**
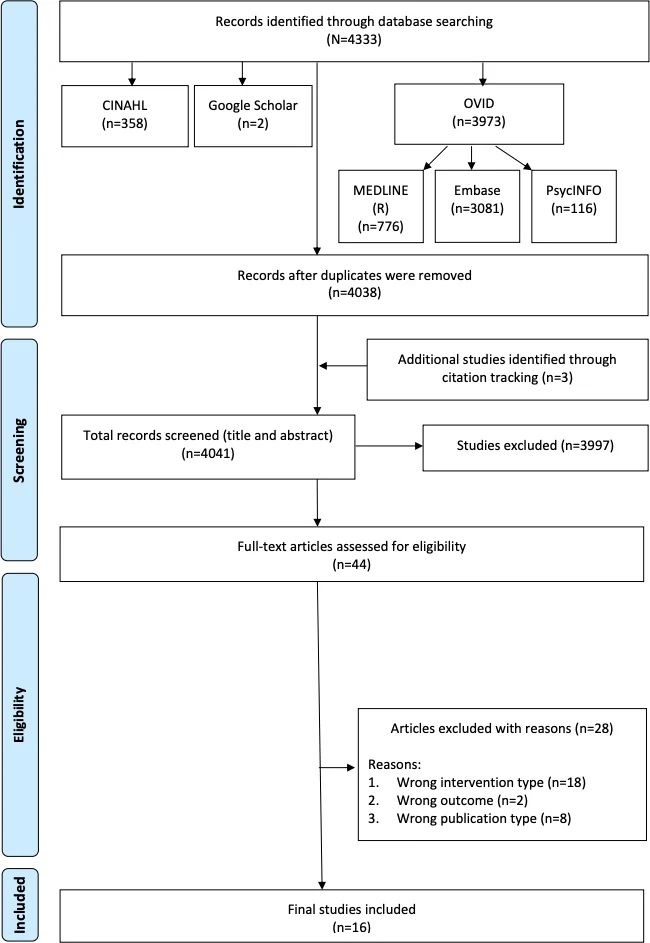
PRISMA (Preferred Reporting Items for Systematic Reviews and Meta-Analyses) flowchart showing the database search detailing the sources used, number of records identified per source, reasons for exclusion, and the number of full-text reports assessed for the final inclusion decision. CINAHL: Current Index to Nursing and Allied Health Literature.

The search identified 4038 citations after removing duplicates (Figure 1), which were retained for abstract screening, and 3 additional papers were identified through backward or forward screening. We also identified 4 studies at the title and abstract screening stage that met our inclusion criteria, but despite our best attempts to contact the authors, full texts were not available.

A total of 3998 papers were excluded due to a lack of relevance to asthma or COPD, or the absence of any digital health component. Subsequently, 44 papers were shortlisted for full-text screening, of which 28 were excluded (18 studies included wrong interventions and stand-alone tools, 8 were the wrong publication type, and 2 reported irrelevant outcomes). Of the included studies, some of the publications were from the same author group, with 3 papers linked to a single trial [32,33,35] and 2 to another RCT [39,42]. Although 1 pilot study [36] was related to a subsequent cohort analysis [37], trial registration numbers and population samples were different, so they were considered as separate studies. Therefore, a total of 16 papers were included in the review, which represented 14 unique studies [29-44].

### Study Characteristics

Of the 16 included papers, 13 investigated asthma only and 2 COPD only [38,41]. One study included both asthma and COPD populations and did not separate these 2, or report individual disease-specific outcomes [43]. Publication years ranged from 2007 to 2024. Eight papers were from the United States [29,31-33,35-37,40], 3 from Canada [30,39,42], 2 from the Netherlands [43,44], and 1 each from Portugal [34], Spain [38], and the United Kingdom [41]. There were 3 RCTs [30,32,34], 5 observational studies [31,33,35,39,41], and 4 quasi-experimental studies [29,38,42,44]. Two were feasibility evaluations [36,40]. One was a cohort trial [37] and another was a cross-sectional study [43]. Details of the reports included are shown in Multimedia Appendix 2.

### Risk-of-Bias in Studies

**Figure 2.**
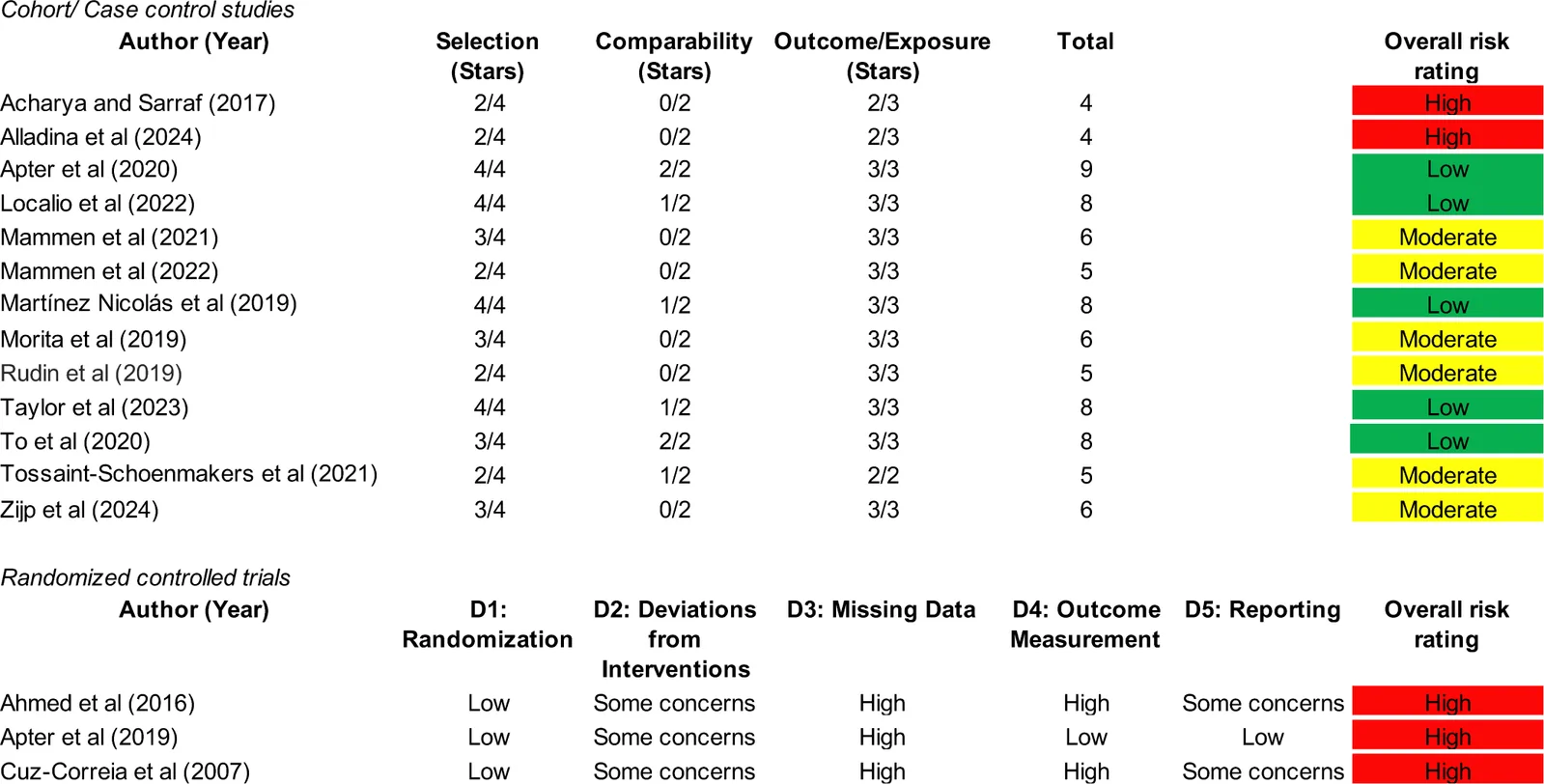
Risk-of-bias rating for the included studies [29-44]. The figure illustrates the risk-of-bias assessment of the included studies with details of the article name, publication year, categories of assessment, and the overall risk-rating decision where high indicates poor quality papers with high risk of bias, moderate indicates medium risk of bias, and low indicates good-quality papers with low risk of bias. The Newcastle-Ottawa Scale (NOS) was used for cohort studies, and an adapted version of the NOS tool was used for cross-sectional studies [48,49]. For NOS screening decisions, a total score of <4 indicates high (red), 5‐6 indicates moderate (yellow), and >7 indicates low risk of bias (green). The Cochrane Risk of Bias Tool 2.0 was used for randomized controlled trials where the overall rating was “high” (red) if any domain was rated high, “low” (green) only if all domains were low, and otherwise “unclear” [50].

In the risk-of-bias quality assessment, 5 out of 16 were rated as high, 6 moderate, and 5 low risk of bias. All of the RCTs received a high risk-of-bias rating due to high attrition and missing outcome data [30,32,34]. Of the other studies, 5 had low risk of bias [1,33,35,38,41], 6 had moderate risk [36,37,39,40,43,44], and 2 had high risk of bias [29,31], mainly due to the absence of a comparator group and a lack of treatment effect assessment against a baseline. Figure 2 details the methodological quality and risk-of-bias decisions for all included papers.

When using GRADE to rate the certainty of domain-level evidence using a narrative summary [52], certainty was judged low to very low for both asthma and COPD, reflecting serious limitations in study design, inconsistency, imprecision, and indirectness (Table 1). Narrative inspection and GRADE assessment suggested variation across studies in terms of their design, population characteristics, intervention scope, and implementation, which may partly account for the inconsistencies in outcomes reported and the differences in findings. Where risk of bias from missing results was suspected, checks of partially published data and related publications were conducted, and authors were contacted to request additional data. Publication bias was not deemed a concern in asthma as studies generally varied in their size, design, geographical representation, and direction of effect, with no consistent pattern of positive findings. In COPD, the findings across all domains were drawn from a limited number of papers, which was considered a publication bias risk. There was also a risk of sample overlap in some of the studies, which was considered when grading the certainty of evidence. For example, Apter et al [32,33] and Localio et al [35] related to the same trial and consisted of the same participants, which may lead to overestimations in the true sample size. The risk of overlap was less clear for Mammen et al [36,37], as the total sample size and group composition (eg, age range) differ, and the authors do not clearly describe whether the participants in the larger cohort trial included the smaller β-test group.

**Table 1.** Certainty of evidence across the quality-of-care outcome domains assessed using the GRADE^a^ approach.

Domains	Studies, n	Report type	Sample size	Risk of bias	Inconsistency	Indirectness	Imprecision	Publication bias	Overall GRADE level of evidence
Asthma^b^									
Effectiveness	10	3 RCTs^c^, 7 non-RCTs	1733 (risk of sample overlap)	Serious concerns	Serious concerns	Not serious	Very serious	Not serious	Very low
Patient-centeredness	11	2 RCTs, 9 non-RCTs	925 (risk of sample overlap)	Serious concerns	Serious concerns	Not serious	Very serious	Not serious	Very low
Efficiency	5	1 RCT, 4 non-RCTs	238 (risk of sample overlap)	Serious concerns	Not serious	Very serious	Very serious	Not serious	Very low
Equity impacts	7	1 RCT, 6 non-RCTs	1108 (risk of sample overlap)	Not serious	Not serious	Serious concerns	Serious concerns	Not serious	Low
Safety	5	1 RCT, 4 non-RCTs	491	Not serious	Serious concerns	Serious concerns	Serious concerns	Not serious	Very low
Timeliness	3	1 RCT, 2 non-RCTs	63 (risk of sample overlap)	Serious concerns	Not serious	Serious concerns	Serious concerns	Not serious	Very low
Chronic obstructive pulmonary disease^d^									
Effectiveness	2	2 non-RCTs	910,222	Serious concerns	Not serious	Not serious	Not serious	Serious concerns	Very low
Patient-centeredness	2	2 non-RCTs	1246	Serious concerns	Serious concerns	Not serious	Serious concerns	Serious concerns	Very low
Equity impacts	2	2 non-RCTs	1246	Serious concerns	Serious concerns	Not serious	Serious concerns	Serious concerns	Very low
Timeliness	1	1 non-RCT	498	Serious concerns	Not serious	Not serious	Serious concerns	Serious concerns	Very low

a GRADE: Grading of Recommendations Assessment, Development, and Evaluation.

b The table summarizes the GRADE assessment for certainty of evidence for asthma. “Domains” refer to the 6 care quality indicators for which pooled effect estimates were not available and only a narrative synthesis of the evidence was provided. Evidence from randomized controlled trials was initially rated as high certainty and observational studies as low certainty, with downgrading applied for risk of bias, inconsistency, indirectness, imprecision, and publication bias, where appropriate. Certainty ratings were categorized as high, moderate, low, or very low, reflecting confidence that the estimated effect is close to the true effect. Concerns were reported as not serious, serious concerns (−1), or very serious concerns (−2). The evidence from the study that reported mixed population (ie, asthma and COPD) without separate outcome measurement was considered applicable to both conditions but with downgrading for indirectness. Where risk of sample overlap has been noted, it relates to studies that potentially used the same sample population for the analyses.

c RCTs: randomized controlled trials.

d Table summarizes the GRADE assessment for certainty of evidence for COPD. “Domains” refer to the 6 care quality indicators for which pooled effect estimates were not available and only a narrative synthesis of the evidence was provided. Evidence from randomized controlled trials was initially rated as high certainty and observational studies as low certainty, with downgrading applied for risk of bias, inconsistency, indirectness, imprecision, and publication bias, where appropriate. Certainty ratings were categorized as high, moderate, low, or very low, reflecting confidence that the estimated effect is close to the true effect. Concerns were reported as not serious, serious concerns (−1), or very serious concerns (−2). The evidence from the study that reported mixed population (ie, asthma and COPD) without separate outcome measurement was considered applicable to both conditions but with downgrading for indirectness. Where risk of sample overlap has been noted, it relates to studies that potentially used the same sample population for the analyses.

### Participants

Study participants were recruited from a range of settings. Five publications were multicenter collaborations involving primary care practices, specialty clinics, pulmonary or ENT departments, and/or academic hospitals [31-33,35,39], and 3 papers were each based in primary care [36,37,42], secondary care [38,41,44], and tertiary care centers [29,30,34]. One study was community-based [43] and 1 ambulatory care–based [40]. The sample size ranged from 7 to 9,09,724 participants [36,38]. Participants were predominantly female (55%-90%) [32,38]. Nine publications reported data on ethnicity [31,32,36-40,42], and the number of White participants enrolled ranged from 0.7% to 78.3% [31,32], whereas the number of Black/African American participants ranged from 9.2% to 76.8% [31,32].

### Patient Portal Functions

Communication options were included in 11 papers using features such as secure messaging options, email alerts, provider logs, emergency contacts or services, and so on [29-31,34,36-39,41,42,44]. Functions supporting health and disease monitoring were reported in 11 papers using asthma diaries, logs, journals, immunization records, wireless spirometry, and so on [29,31-34,36,37,39,42,44]. Self-management support and educational components using personalized action plans, social forums, training, and linked resources were mentioned in 9 papers [29-31,34,36,37,41,42,44]. Seven papers included provider-facing dashboards or decision support tools [30,31,34,36,37,41,44]. Appointment management options (eg, review, scheduling, and reminders) were reported in 4 papers [32,33,35,38], and 5 papers offered visual care indicators using features such as disease trend charts, traffic light displays, and so on [31,34,39,42,43]. Three papers included medication lists or review and repeat prescription order functions [32,33,35]. Medication management tools using inhaler logs and trackers appeared in 2 papers [29,31]. One study also offered options to customize monitoring schedules via a clinician dashboard [34]. Six out of the 16 papers offered access to provider records, which mostly consisted of structured data such as laboratory results, action plan updates, clinical decision support outputs, and so on [32,33,36-39,42,43]. Data on quality outcomes related to specific functions were not examined in sufficient detail by any of the included studies to support meaningful conclusions regarding the effects of individual features.

### Comparators

Comparators were patients receiving regular care [30,42], portal access but without home visits [32,33], paper diaries and other web-based tools or educational material [34], nonusers of the intervention [41], and English versus Spanish-language speakers [35].

### Outcomes

**Figure 3.**
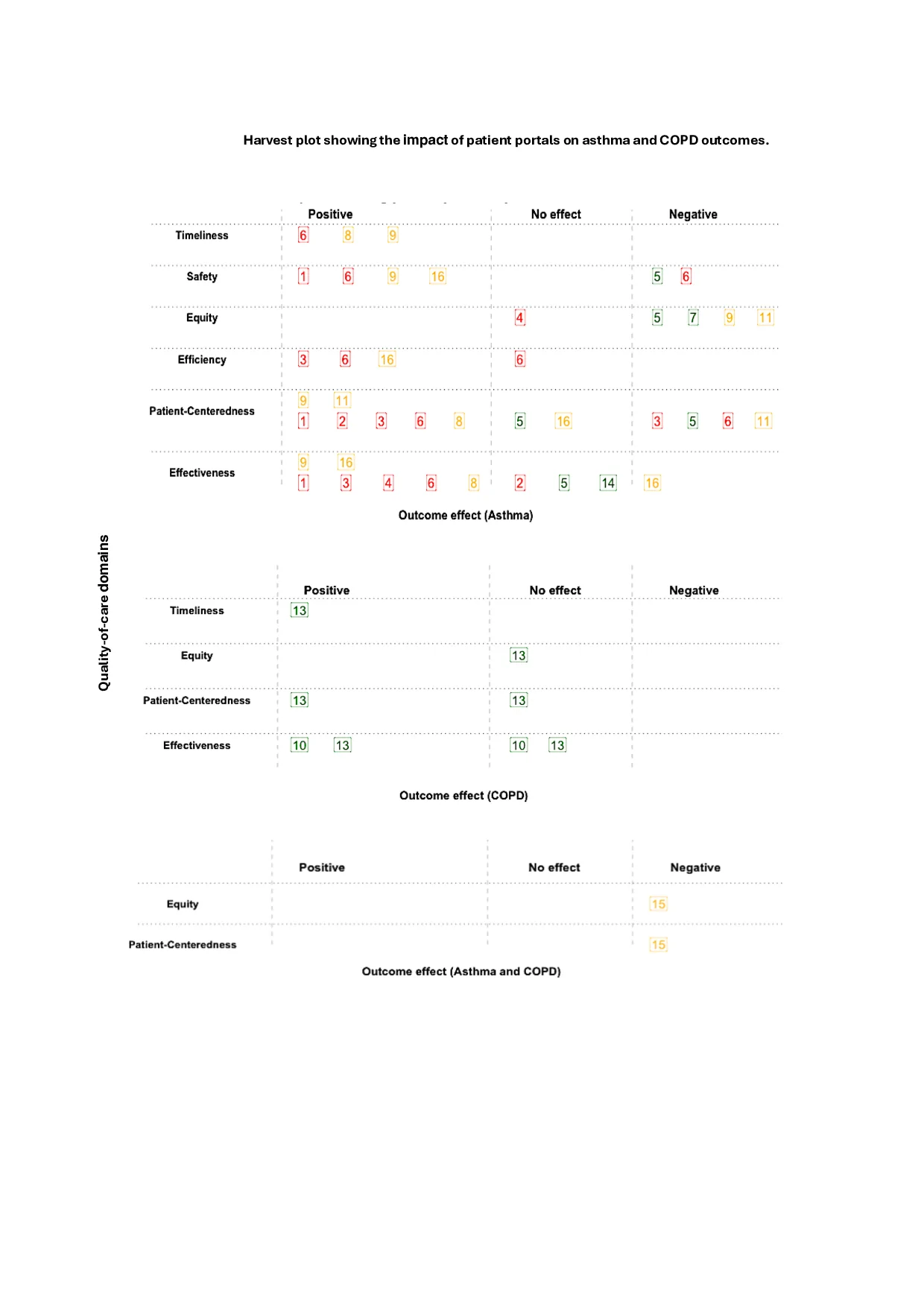
The figure illustrates the outcome effects, categorized as “Positive,” “No effect,” and “Negative” related to the specific domain and their indicators. Multiple outcomes of the same category were counted once, and studies with different outcome results were recorded separately. The box color represents risk-of-bias rating, where red indicates high risk of bias, yellow indicates medium risk of bias, and green indicates low risk of bias. COPD: chronic obstructive pulmonary disease. Studies: (1) Acharya and Sarraf (2017) [29]; (2) Ahmed et al (2016) [30]; (3) Alladina et al (2024) [31]; (4) Apter et al (2019) [32]; (5) Apter et al (2020) [33]; (6) Cruz-Correia et al (2007) [34]; (7) Localio et al (2022) [35]; (8) Mammen et al (2021) [36]; (9) Mammen et al (2022) [37]; (10) Martinez Nicolas et al (2019) [38]; (11) Morita et al (2019) [39]; (12) Rudin et al (2019) [40]; (13) Taylor et al (2023) [41]; (14) To et al (2020) [42]; (15) Tossaint-Schoenmakers et al (2021) [43]; and (16) Zijp et al (2024) [44].

There were differing numbers of publications across different quality domains: effectiveness (12 papers), patient-centeredness (12 papers), equity (8 papers), efficiency (5 papers), safety (5 papers), and timeliness (4 papers). Details of the outcomes for the individual studies are presented in Table 2, and Figure 3 shows a harvest plot with intervention effects in accordance with the SWiM guidance [26].

**Table 2.** Summary of reported outcomes categorized according to the 6 quality-of-care domains. The table provides a summary of the outcomes categorized according to the 6 quality-of-care domains of effectiveness, patient-centeredness, efficiency, equity, safety, and timeliness among adults with asthma and chronic obstructive pulmonary disease using patient portals. Could all RCTs in the table have the superscript **c**, to make clear they all refer to “Randomised Controlled Trial”?

Author (year)	Effectiveness	Patient-centeredness	Efficiency	Equity impact	Safety	Timeliness
Acharya and Sarraf (2017) [29]	Health care service use: 83% of health care providers reported that the use of the Pro-Care application reduced the number of office visits for patients by over 60%.	Self-management: The majority of the patients strongly agreed that the real-time feedback was very useful in managing their asthma condition. Most patients found the application helpful in keeping track of their prescribed action plans. Satisfaction: The majority of the patients found the application to be successful in asthma management.	Not stated	Not stated	Security: The IT group reported no instances of instability or unavailability during the evaluation period.	Not stated
Ahmed et al (2016) [30]	Disease control: No significant between-group differences over time in asthma control status.	QoL^a^: Between baseline and 3 months, only the intervention group showed a significant improvement in MAQLQ^b^ score (mean change 0.67, 95% CI 0.36-0.98). No significant differences between 3 and 6 months (mean change −0.01, 95% CI −0.35 to 0.32) and 6‐9 months (mean change −0.12, 95% CI −0.46 to 0.22) for the intervention group. Also, no significant differences over time for the control group and between 2 groups. MAQLQ significantly associated with improvement in depression PHQ^c^ (mean change −0.27, 95% CI −0.37 to −0.18 for 5 unit change), self-efficacy (mean change 0.24, 95% CI 0.16-0.32) and ACT^d^ score (mean change −0.25, 95% CI −0.30 to −0.20).	Not stated	Not stated	Not stated	Not stated
Alladina et al (2024) [31]	Disease control: Asthma status was consistent and reported as “good” in >69% of daily entries. Overall, 14% of patients experienced asthma exacerbations. Medication use: Overall, 76% of patients reporting “bad” asthma status used quick relief or rescue medication.	Satisfaction: Overall, 66% of patients were satisfied or very satisfied with the app. Overall, 49% rated the app “excellent,” 30% “good.” Overall, 71% of HCPs^e^ reported the app as “very easy” to implement, and 29% reported that the app “moderately” or “slightly” helped patient management. Recommendation: Overall, 66% would likely or very likely recommend the app. Engagement: Overall, 47% did not use the asthma plan, 52% skipped appointment scheduling, 57% skipped education, and 65% did not message the clinic.	Streamline of communication and appointments: 44% felt that the app helped discuss asthma with care professionals; 37% said it made appointments smoother.	Not stated	Not stated	Not stated
Apter et al (2019) [32]	Disease control: Intervention: 27.8% (42 patients) achieved asthma control (ACQ^f^ ≤1.5) at 12 months. Control: 23.3% (35 patients) achieved asthma control at 12 months. Intervention: 25% (37 patients) had a clinically important improvement (0.5-point reduction in ACQ). Control: No patients had clinically important improvement. Health care service use: Only hospitalizations showed statistically significant improvements in the intervention group at 12 months (−0.53, 95% CI −1.08 to −0.024).	Not stated	Not stated	Intervention benefit: Spanish as the primary language and trust on the internet and the patient portal for clinical information were possible effect modifiers, but these subgroups did not prove to have different benefits for the portal plus home visit intervention.	Not stated	Not stated
Apter et al (2020) [33]	Disease control: No association between portal usage rates and asthma control at 12 months compared with baseline.	QoL: No association between portal usage rates and asthma quality of life at 12 months compared with baseline. Satisfaction: 62% had little confidence that the portal could improve communication with their doctor.	Not stated	Portal use: Patients with chronic diseases or past hospitalizations were less likely to use the portal, but it was unrelated to asthma severity. Overall, 50% of potential participants had computer access, but some chose not to use the portal.	Privacy: Overall, 16% expressed concerns about portal confidentiality.	Not stated
Cruz-Correia et al (2007) [34]	Disease control: More participants in the intervention group believed that the platform could improve their asthma control (73% vs 50%), treatment adherence (64% vs 53%), and asthma care (80% vs 69%) in the intervention versus paper diary group.	Satisfaction: More patients rated the intervention as very useful compared with the paper diary (*P*=.038). Viewing previous data was considered much easier with the Internet tool than with paper (*P*=.038). Overall, 92% were very interested in using the intervention in the future versus 12% for paper tools (*P*=.002). Adherence dropped after the second visit, especially after weeks 1 and 4. Paper diary showed higher recorded adherence (*P*<.001), but likely overestimated.	Streamline of tasks: The median time to complete both internet and paper diaries was similar (3 minutes per entry; *P*=.675). PEF^g^/FEV_1_^h^_1_ monitoring took less time (median 2 minutes; *P*=.028 for internet and *P*=.036 for paper).	Not stated	Safety report: No safety concerns reported. Authors conclude that the technology was considered safe. Issues related to internet connection (n=9), user interface (n=5), system errors (n=3), and question interpretation (n=2) were reported.	Reporting timeline: Paper tools lacked timeliness; users often delayed entries (some admitted bulk-filling days later).
Localio et al (2022) [35]	Not stated	Not stated	Not stated	Portal use: Overall, 42% (n=102) of English speakers did not use the portal vs 56% (n=29) of Spanish speakers (Pearson chi-square=3.84; *P*=.05). In total, 49% (n=32) of low-literacy English speakers did not use the portal vs 69% (n=18) of low-literacy Spanish speakers (Pearson chi-square=10.4; *P*=.02). Overall, 25% of English-speaking participants reported barriers to portal use, compared with 76% of Spanish-speaking respondents. Access and knowledge: In response to “I would use the internet more if...” Overall, 25% of English speakers (out of 182 respondents) and 26% of Spanish speakers (out of 43 respondents) selected “I knew more/learned how.” In total, 25% of English speakers (n=182) and 23% of Spanish speakers selected "I had access”	Not stated	Not stated
Mammen et al (2021) [36]	Disease control: 1.5 (SD 1.02) point mean ACQ improvement in asthma control (CI^i^ 0.59‐2.51; *P*=.007) at 3-month follow-up. Significant improvements were seen in morning symptoms, nighttime wakening, activity limitations, and shortness of breath, with greatest effects on reductions in wheezing with mean 1.87 (SD 1.34) (*P*=.011, CI 0.61-3.10). 14.86% (SD 19.4) mean increase in FEV1%_pred_^j^ (CI −3.09 to 32.80l;. *P*=.089). Health service use: Preventive health care usage increased significantly (1.86 visits per year vs 0.29 per year prior, CI 0.67-2.47; *P*=.005). Medication use: Increased prescriptions for controller medications (9.29 prescriptions per year vs 1.57 prescriptions per year, CI 4.85-10.58; *P*=.001).	QoL: 1.91 (SD 1.53) point mean improvement (*P*=.016, CI 0.50-3.31) evenly distributed across all domains (symptoms, activity limitations, emotional functioning, and environmental stimuli). Satisfaction: 93.9%	Economic evaluation: $186.52 per person. Nursing time: 44.47 minutes per visit.	Not stated	Not stated	Time to care: 100% care provider response to follow-up, and home symptom monitoring facilitated timely care.
Mammen et al (2022) [37]	Disease control: 80% participants had uncontrolled asthma at baseline and at 6 months, 80% had well-controlled asthma. Patients with worse asthma control at baseline benefited the most from the intervention, with a strong correlation between baseline ACQ and symptom improvement (*r*=-0.82, *P*<.001). FEV1%_pred_ increased by 4.2% Medication use: Adherence increased from 45.58% to 85.29% (CI 14.79-64.62). Guideline-based care: HCP adherence to the guideline-based therapy increased from 43.3% at baseline (CI 22.11-64.55) to 86.7%.	QoL: Improvement in QoL was strongly associated with improved control (*r*=0.80; *P*<.001) but not with FEV1%_pred_ (*r*=0.087; *P*=.648). Satisfaction: 95.7% acceptability. 29/30 patients and all providers indicated the intervention worked better than usual care.	Economic evaluation: $186.52 per person over the 6-month period.	Wider health determinants: Improvements in FEV1%_pred_ were greatest for smokers (+10.27% vs nonsmokers+0.68%, CI 1.72‐17.45), males (+11.27% vs females +0.11%, CI 3.62‐18.72), and those with high-school education or less (+7.94% vs any college education −0.071%, CI 0.20‐15.82). No other significant differences in intervention effects based on gender, smoking status, education, race or ethnicity, or presence of comorbid mental illness.	Technical issues: 93.2% of visits showed no disruptive technical issues.	Time to care: 38% of visits occurred after-hours or on weekends.
Martínez Nicolás et al (2019) [38]	Health service use: For the COPD^k^ group, there was a significant decline in slope for any hospitalizations per 10,000 patients per month (before EPP^l^=1.33, SE 0.3, *P*<.001 vs after=−4.38 SE 0.66; *P*<.001) and emergency department use (before=2.27, SE 0.45, *P*<.001 vs. after=−5.08, SE 1.26; *P*<.001) and any outpatient service use (before=0.002, SE 0.0002; *P*<.001 vs after=−0.004, SE 0.001; *P*<.001) but no change in 30-day hospital readmissions (before=0.33, SD 0.10; *P*<.05 vs after=−1.07, SD 0.59).	Not stated	Not stated	Not stated	Not stated	Not stated
Morita et al (2019) [39]	Not stated	Satisfaction: 63.8% (74/116) of the patients agreed or strongly agreed that the intervention was helpful in the management of their asthma. Patients agreed or strongly agreed that the intervention was helpful in the management of their asthma. 65.2% (75/115) of the patients were confident that the intervention was correct when it presented the patient’s asthma action plan zone of control. Patients were confident that the intervention was correct when it presented the patient’s asthma action plan zone of control. 49.6% (58/117) of the participants agreed or strongly agreed that they would continue to use the intervention after the study. Participants agreed or strongly agreed that they would continue to use the intervention after the study. The platform scored 71.1 (SD 19.9) on the standardized SUS^m^ at 12 months indicating good usability. Engagement: Declined use within the first 4 weeks. Overall, 67.5% (83/123) of the participants used the platform weekly initially and only 57.7% (71/123) used the platform in week 45.	Not stated	Use: Age (≥50 years) was associated with higher usage. 55.2% (76/138) of the participants had a smartphone and reported being comfortable or very comfortable with its use, but post hoc analysis did not find an association between platform usage and having a smartphone.	Not stated	Not stated
Rudin et al (2019) [40]	Not stated	Engagement: On average, 25% of all questionnaires had results severe enough to qualify for a call back from a nurse; however, 77% declined at least 1 option for call back.	Not stated	Program completion: Female patients had significantly higher weekly questionnaire completion rates than males (88 vs 73%; *P*=.02). Patients with a bachelor’s degree level or higher completed significantly more questionnaires than those with lower education (94% vs 74%; *P*<.01). Clinical characteristics, age, and ethnicity were not significantly associated with questionnaire completion.	Not stated	Not stated
Taylor et al (2023) [41]	Health service use: Reduction in annual COPD or respiratory-related admissions (mean change=1.21days in intervention group vs 0.62 days in control) and bed-occupied days (mean change=8.12 days in intervention group vs 3.38 days in control). Mortality: Lower 12-month mortality rate in intervention vs control (16.9% vs 24.1%), but this difference was not statistically significant (*P*=.215).	QoL: Health-related QoL (measured via EQ-5D visual analogue scale) remained broadly stable over the course of the study. Disease burden: Symptom burden (measured via COPD assessment test) also showed no significant change during the study period. Self-management: Median self-managed exacerbations were 2 per year for the intervention group with a higher median exacerbation of 4 per year in patients with the highest application usage.	Not stated	Use: Overall, 77% of the participants were sustained use. Subgroup analysis of participants resident in more socioeconomically deprived postcode areas revealed equivalent usage to the rest of the cohort.	Not stated	Time to adverse events: Median time to first COPD or respiratory–related admission or death was longer in the intervention cohort (335 days vs 155 days; unadjusted hazard ratio=0.740, 95% CI 0.550-0.996; *P*=.047).
To et al (2020) [42]	Health service use: No significant differences between the Breathe group, non-Breathe group, and population controls in hospitalizations, ED visits, and physician office visits.	Not stated	Not stated		Not stated	Not stated
Tossaint-Schoenmakers et al (2021) [43]	Not stated	Self-efficacy: Higher education levels were associated with lower self-efficacy in managing health; *P*<.10 (*b*=−3.521, 95% CI 6.469 to −0.572; *P*=.02)	Not stated	Use: Higher age and high education were associated with decreased usability: respectively, *b*=−0.094, 95% CI 1147 to −0.042 (*P*<.001); and *b*=−2.512, 95% CI 4.791 to −0.232 (*P*=.03). Patients with asthma or COPD scored significantly lower than those with no chronic disease (*b*=−3.630, 95% CI −6.545 to −0.715; *P*=.02).	Not stated	Not stated
Zijp et al (2024) [44]	Disease control: Asthma control improved postintervention (ACQ-6-score: baseline μ=2.1, SD=1.3; postintervention μ=1.3, SD=1.0; *t*=2.61, *P*=.02, d=0.70) Medication use: No significant change in rescue medication use (Last wk additional medication: baseline μ=2.0, SD=1.5; postintervention μ=1.4, SD=1.1; *t*=-1.63, *P*=.10, *d*=-0.06)	QoL: No significant changes in asthma-related quality of life trends RIQMON-10^n^ score: μ=22.6, SD=6.9; postintervention μ=18.9, SD=7.7; *t*=1.70, *P*=.11, *d*=0.46. EQ-5D-5L score: μ=1.8, SD=0.7; postintervention μ=1.6, SD=0.6; *t*=1.75, *P*=.10, *d*=0.47. VAS-QoL^o^ score: Baseline μ=53.5, SD=17.5; postintervention μ=67.4, SD=18.9; *t*=-2.08, *P*=.06, d=0.56. Self-management: Perceived ability to manage asthma remained stable with no significant change (*P*=.84) (PCAQ^p^ score: baseline μ=21.4, SD=2.7; postintervention μ=21.6, SD=5.0; *t*=−0.231, *d*=0.06).	Economic evaluation: Indirect costs showed no significant change iPCQ^q^ score: Baseline μ=34.8, SD=54.7; postintervention μ=22.8, SD=53.3; *t*=1.057, *P*=.33, *d*=0.37. iMCQ^r^ score: Baseline μ=4.6, SD=3.7; postintervention μ=2.9, SD=3.0; *t*=1.52, *P*=.15, *d*=0.41.	Not stated	Adverse events: No adverse events reported by patients or providers.	Not stated

a QoL: quality of life.

b MAQLQ: Mini-Asthma Quality of Life Questionnaire.

c PHQ: Patient Health Questionnaire.

d ACT: asthma control test.

e HCPs: health care providers.

f ACQ: Asthma Control Questionnaire.

g PEF: peak expiratory flow.

h FEV_1_: forced expiratory volume in 1 second.

i CI: confidence interval reported as specified in the original study. Where the confidence level was not stated, no level was assumed.

j FEV1%_pred_: forced expiratory volume in 1 second percentage predicted.

k COPD: chronic obstructive pulmonary disease.

l EEP: electronic patient portal.

m SUS: System Usability Scale.

n RIQMON-10: Respiratory Illness Questionnaire-Monitoring 10.

o VAS-QoL: Visual Analogue Scale–Quality of Life.

p PCAQ: Perceived Control of Asthma Questionnaire.

q iPCQ: iMTA Productivity Cost questionnaire.

r iMCQ: iMTA Medical Consumption Questionnaire.

#### 1. Effectiveness

Overall, evidence from the 12 studies on asthma and COPD suggests possible improvements in selected indicators of effectiveness, including asthma control, medication adherence, and health care usage. However, findings were inconsistent and often derived from interventions that included additional support beyond portal use. Evidence for COPD was restricted to 2 observational studies, with reported hospitalization reductions but no mortality benefits. GRADE assessments showed that the certainty of evidence was very low for both asthma and COPD.

##### Asthma

Seven out of the 10 studies on asthma found evidence of improvements in at least one of the indicators of effectiveness [29,31,32,34,36,37,44]. Four studies showed either no difference in disease control [30,33], health care service use [42], or small, nonsignificant gains in rescue medication use and Asthma Control Questionnaire (ACQ) scores [44]. Among the studies with positive effects, improved disease control was noted in a large RCT rated as high risk of bias, which showed a modest increase in well-controlled asthma (27.8% intervention vs 23.3% control) and clinically significant improvements (0.5-point ACQ reduction in 25% intervention group) at 12 months of follow-up [32]. However, a subsequent paper by the same group found that disease control improvements were unrelated to portal usage rates [33]. In other smaller studies with moderate bias risks, a 1.5-fold improvement in ACQ scores at follow-up was noted (2.1 baseline vs 1.3 postintervention) [44]. There were also improvements in guideline-based care (43.3% at baseline vs 86.7% at 6 months) and an inverse correlation between baseline disease control and symptom improvements (*r*=−0.82; *P*<.001) reported in a small cohort trial, suggesting that patients with the poorest asthma control may benefit more from the intervention [37].

Assessment of medication and health care service use in studies with moderate bias risks showed some evidence of improvements in medication adherence rates (45.6% at baseline to 85.3% postintervention) [37], greater medication requests, and more preventive health care visits [36]. Reductions in hospital visits were also noted in a majority of provider reports (>60%) in Acharya et al [29], which was rated as high risk of bias. In another RCT with risk of bias, hospitalization rate reduction at 12-month follow-up was greater among patients who received training in portal use alongside home visits than among patients with no home visit support [32].

##### COPD

In the 2 COPD studies, reductions in the monthly hospitalization trend, emergency visits, and outpatient appointments postintervention were reported in a large quasi-experimental trial with low risk of bias (slope change −4.38,−5.08, and −0.004 per 10,000 patients per month, respectively), but there were no changes in hospital readmissions [38]. A greater mean change in annual hospital admission days (1.21 vs 0.62) and occupied bed days (8.12 vs 3.38) was reported in intervention versus control in another low-bias risk observational study, suggesting more effective disease management [41]. However, there were no survival benefits as the difference in the 12-month mortality rates was nonsignificant (16.9% intervention vs 24.1% control; *P*=.22) [38]. The findings were based on only 2 observational studies and they should be interpreted cautiously.

### 2. Patient-Centeredness

Overall, evidence from the 12 studies on asthma and COPD indicates high levels of patient satisfaction. Although some short-term QoL improvements among patient portal users with asthma were noted, sustained engagement was limited. Although QoL improvements remained relatively stable in COPD, this evidence was drawn from only 1 observational study. GRADE assessments showed that the certainty of evidence was very low for both asthma and COPD.

#### Asthma

Out of the 11 papers reporting on patient-centeredness in asthma, 7 papers showed positive effects such as high patient satisfaction [29-31,34,36,37,39], but issues related to adherence and engagement were also identified. These studies ranged from high to moderate risk of bias. Satisfaction rates were as high as 93.9% in a pilot study by Mammen et al. (2021) [36]. While some studies showed that patients rated patient portals better than usual care or paper diaries [34,37], sustained use was a notable issue with a drop in usage within the first month [34]. Similar issues with long-term engagement were also reported in a medium risk-of-bias rated observational study by Morita et al [39], with just over half of the participants (57.7%) using the tool at week 45, although the majority (65.2%) trusted the intervention. Alladina et al [31] also reported that despite 66% of patients recommending the application and nearly half (49%) rating it as excellent, overall usage rates remained low. In Apter et al [33], most patients (62%) expressed low confidence that patient portals would improve provider communication. Also, Zjip et al [44] showed that perceived self-management, as measured by the Perceived Control of Asthma Questionnaire, remained unchanged postintervention (21.4-21.6; *d*=0.06; *P=*.84), suggesting that the platform had a limited impact on patient self-management perceptions.

Five papers showed modest but variable QoL improvements [30,33,36,37,44]. A small study by Zjip et al [44] used the EuroQol questionnaire incorporating a visual analogue scale and found a nonsignificant QoL increase from 53.5 (SD 17.5) to 67.4 (SD 18.9) points (*d*=0.56; *P*=.06). The Mini Asthma-Related Quality of Life Questionnaire was used by all other studies. Although Ahmed et al [30] reported short-term QoL improvements at 3 months in the intervention group (mean Mini Asthma-Related Quality of Life Questionnaire change=0.67, 95% CI 0.36-0.98), this study had high risk of bias and they found no changes at 6 and 9 months. All other studies were rated as moderate risk of bias, except Apter et al [33], which had a low risk of bias, and they found no long-term QoL change at 12 months [33]. Also, Mammen et al [37] found that asthma-related QoL improvements were related to asthma control only (*r*=0.80; *P*<.001) but not with lung function measured using FEV1%_pred_ (*r*=0.087; *P*=.648), suggesting that QoL changes were linked to subjective asthma control but not objective lung function measures.

#### COPD

In COPD, the observational study by Taylor et al [41] showed that health-related QoL was stable over time, and symptom burden remained unchanged. Engagement was higher in patients experiencing more disease exacerbations, with an average of 4 self-managed exacerbations annually compared with the overall group average of two [41]. Evidence related to asthma or COPD showed that the presence of respiratory disease was not significantly associated with self-efficacy, and the outcomes for the 2 respiratory conditions were not presented separately [43].

### 3. Equity

Overall, evidence from the 8 studies on asthma and COPD found that patient portal use may be influenced by a range of sociodemographic and access-related factors, including technology availability, language, and digital literacy. Evidence of subgroup differences was limited and inconsistent, but access barriers appeared to affect different user groups. GRADE assessments showed that the certainty of evidence was low for asthma and very low for COPD.

Also, nearly half of the papers excluded participants without access to technology or the internet [31,34,37,39,40,42,44], and 2 papers explicitly focused on younger age groups below 40 years or 45 years [36,37]. Although some papers recruited purposeful samples of participants from areas of high deprivation [32,33], and reported on differences by language [35], exclusion of individuals without internet access or digital skills limits the generalizability of findings.

#### Asthma

Six out of 8 papers with medium to low risk of bias reported asthma-related subgroup differences in patient portal access and use, but the indicators used varied significantly [32,33,35,37,39,40]. One small cohort study showed greater lung function improvements (FEV1%_predicted_) among smokers, males, and participants with a high school education or less [37]. In terms of compliance, females and participants with a minimum bachelor’s degree had higher questionnaire completion rates, showing better engagement in a feasibility trial, while age, clinical characteristics, and ethnicity were unrelated [40]. Spanish speakers, particularly those with lower literacy, were found less likely to use patient portals than English speakers in a study with low risk of bias (56% vs 42%; low-literacy groups=69% vs 49%). However, nearly a quarter in both groups reported that they would use the internet more if they had more knowledge or had access (English vs Spanish speakers=25% vs 26% and 25% vs 23%, respectively), highlighting access barrier issues among all participants [35]. In contrast, Apter et al [32] found no significant differential benefit from the patient portal with home visit across any patient subgroups, including participants with different primary languages (Spanish vs English) [32]. In an observational study, limited access and personal choice were reported as factors influencing technology engagement, as patients with chronic illness or prior hospitalizations were found less likely to use the portal [33]. A larger observational study with medium risk of bias by Morita et al [39] showed that older age (50 years and older) was associated with higher portal use, although only half of all participants reported smartphone ownership and use (55.2%) [39].

#### COPD

One COPD paper by Taylor et al [41] showed no subgroup differences in patient portal use between participants from socioeconomically deprived and nondeprived areas [41]. Tossaint-Schoenmakers et al [43] showed lower portal usability in those with asthma or COPD than in those with no chronic disease (*b*=−3.630; *P=*.02) and among those with higher education and age, suggesting the need for adaptive implementation strategies.

### 4. Efficiency

Overall, evidence from 5 studies on asthma suggested that patient portals may offer perceived improvements in aspects of care efficiency, such as enhanced communication and streamlined clinical interactions. However, these findings were based on subjective measures reported by small-scale studies, with limited objective evaluation of resource usage or cost-effectiveness. GRADE assessments showed that the certainty of evidence was very low for asthma.

Three out of the 5 papers showed positive impacts on asthma care [31,34,44], and communication, where 44% of participants in Alladina et al [31] reported improved disease-related discussions with their care providers, and 37% agreed that it helped their appointment run more smoothly. In terms of task completion, the feasibility of internet diary entry was rated similar to paper diaries, while lung function monitoring was faster (median 2 minutes) [34]. However, the overall evidence was considered weak due to high risk of bias.

Economic evaluation using descriptive metrics showed a mean cost of the patient portal of US $186.52 per participant over 6 months and an average nursing time of approximately 45 minutes per visit [36,37]. However, these findings were from studies conducted in the United States only and objective cost-effectiveness and resource usage outcomes were rarely assessed.

### 5. Safety

Overall, 5 papers included safety reports and all were on asthma. Evidence indicated that patient portals were generally perceived as safe, with no reports of significant adverse events. However, the absence of robust and standardized safety assessments limits confidence in these findings. GRADE assessments showed that the certainty of evidence was very low for asthma.

Four out of the 5 studies with high to moderate risk of bias reported positive effects with no safety-related concerns noted by the participants or by the group involved in technology implementation [29,34,36,44]. There were also no adverse events reported in a small experimental study by Zijp et al [44]. Privacy concerns were reported by 16% of participants in Apter et al [33], and Cruz-Correia et al [34] noted that internet connectivity issues were most frequently reported.

### 6. Timeliness

Overall, evidence from the 5 studies on asthma and COPD found that patient portals may support more timely care processes, including faster symptom reporting and improved communication in asthma. However, these findings were based on a small number of studies with varying designs and outcomes. In particular, only 1 observational study reported on COPD, and it noted longer time to hospitalization. GRADE assessments showed that the certainty of evidence was very low for both asthma and COPD.

#### Asthma

Three papers reported on timeliness in asthma and they demonstrated some positive effects [34,36,37]. Functions such as remote symptom logs–facilitated timely care were reported in a small pilot study, and patients received 100% response from care providers to follow-up requests [36]. In an RCT with high risk of bias, Cruz-Correia et al [34] showed that patient portals reduced symptom-reporting delays and eliminated bulk entry errors attributed to paper-based tools.

#### COPD

A large observational study rated as low risk of bias by Taylor et al [41] reported on time to adverse events in COPD and found that the patient portal group went longer without COPD or respiratory-related hospitalization or death than controls without patient portal access (335 days vs 155 days), with a 26% lower risk in the intervention group (hazard ratio 0.74; *P=*.047).

## Discussion

### Interpretation and Main Findings

This systematic review found that the current evidence base in asthma and COPD remains methodologically weak and insufficient. Evidence on effectiveness showed some increases in disease control, medication adherence, and preventive service use, but limited evidence on long-term health benefits and survival was noted. Findings related to patient-centeredness revealed high satisfaction and some quality-of-life gains, but this did not translate to sustained engagement or clinical benefits. We also noted exclusion of participants experiencing digital health disparities and access barriers. When assessed collectively, the body of evidence across the IOM domains showed an overall high risk of bias, imprecision, and inconsistencies, resulting in low to very low certainty. Evidence on COPD is particularly limited.

This review adds to the existing literature by synthesizing available studies on the specific impacts of patient portals on quality of care for asthma and COPD. Although clear positive effects (eg, improvements in care through patient portal use) were not identified, these findings remain important in highlighting the current lack of strong evidence and identifying priorities for future research and quality-of-care improvements. This review cautions against a reliance on patient portals for clinical management of asthma and COPD and highlights that more methodologically robust primary studies are needed to identify which portal functions may yield clinically meaningful benefits for patients with respiratory disease.

### Comparison With Existing Literature

We captured a modest number of papers on patient portal use in asthma and COPD (n=16), which highlights an emerging body of evidence, particularly for asthma, which was reported in the majority of the studies included. However, comprehensive evaluation of their impacts across defined quality domains for both conditions remains sparse, reflecting broader concerns that the implementation of health technologies may be outpacing the evidence supporting their benefits [53]. In asthma, evidence of positive disease control is in line with a previous review of wider digital health interventions, which found benefits in symptom management, medication adherence, and reduced emergency visits [18]. Although we also noted some benefits in markers of effectiveness, such as improved provider care and greater symptom reductions in those with the poorest baseline control, these advantages coincided with cosupport strategies such as home visits and portal use training. Facilitation strategies such as structured onboarding, technical support, along with provider encouragement are established best practices in patient portal implementation and drivers of sustained engagement [24,54], and our findings indicate that they may remain important. However, this evidence is primarily driven by methodologically weak studies and must be interpreted with caution.

The evidence on patient-centeredness also varied, but patient portal satisfaction was generally high. However, this did not translate to sustained use as the attrition rate remained high in both short-term (ie, 1 month) and long-term follow-up. These patterns suggest an initial positive patient response to patient portals but with challenges realted to continued engagement, a trend also reported in the wider digital health literature [55,56]. This could be due to reasons such as lack of motivation or usability issues, which need to be further investigated [56]. In asthma, QoL improvements were limited to the short term and not sustained over longer periods, but it remained unchanged in COPD. However, the limited evidence on COPD restricts the generalizability of findings and the strength of evidence.

In terms of equity, we found a notable exclusion of individuals with low technology literacy and access, reinforcing the evidence of underrepresentation of certain disadvantaged populations in digital health studies, which is a known concern [57]. Exclusion of certain user groups may have biased the study findings and led to an overestimation of the effectiveness of these interventions. Although some of the studies we reviewed recruited a purposeful sample of marginalized groups and reported some access inequalities and usage barriers, these issues appear to have affected both disadvantaged and nondisadvantaged populations equally [35]. Therefore, disparities in digital health may persist across diverse populations, requiring concerted efforts to improve accessibility and engagement [58]. Notably, 1 paper showed lower patient portal usability among individuals with respiratory disease and with higher education [43]. Although people with a higher disease burden are more likely to use technology to manage their health [59], our findings suggest that clinical need alone does not drive uptake, and education does not guarantee digital competency. This complexity is acknowledged in the literature, which highlights how multiple factors such as prior technology exposure, personal motivation, and support availability can interact to affect digital access, literacy, and skills needed to effectively engage with health care technologies [60,61]. Therefore, the need to involve participants from different backgrounds at every stage from research through to delivery and to coproduce interventions with them may be important to ensure that technological solutions are equitable and truly responsive to the needs of different user groups [62].

Regarding safety, limited evidence showed that patient portals were generally considered safe, with some reports of privacy concerns in asthma. Previous reviews highlight that security and privacy are key barriers restricting successful uptake of interventions providing EHR access, but we could not find strong evidence to support this [18,63,64]. However, this evidence was derived from studies with low to moderate methodological quality and includes small-scale, pilot trials, so the observed lack of association may be due to study limitations instead of an actual absence of safety concerns.

### Strengths and Limitations

This study systematically synthesizes diverse evidence from studies using varied quantitative methods, endpoints, and geographical contexts using the IOM framework for a structured comparison. To the best of our knowledge, this is among the first studies to provide a comprehensive overview of the current state of evidence on patient portals in asthma and COPD by focusing on multiple health care quality domains. We conducted a narrative synthesis in line with SwiM guidance, grouping outcomes according to the IOM quality-of-care domains. This required subjective judgment, particularly where studies reported overlapping or nonstandardized outcomes, which need to be considered when interpreting results. We combined PRISMA and SWiM methods alongside the GRADE approach to assessing evidence certainty. All of these processes require interpretation of data and findings, and these may differ across groups. Nonetheless, we provide access to the relevant documentation in the appendices to aid transparency.

There are also other important limitations that warrant careful consideration. First, the current evidence is driven by small, often underpowered feasibility studies. Particularly for COPD, there is a lack of substantial evidence and high-quality studies. Although we conducted a comprehensive literature search using a broad set of key terms, it is possible that we may have missed some EHR-linked patient portal interventions used in asthma and COPD, which are not specifically indexed as such, or because they were not written in English. Also, all included studies were published post-2007, likely reflecting the broader adoption of patient portals from 2006, initially in primary care and outpatient settings [24]. As the use of digital health technologies appears to be increasing, especially for asthma and COPD, more evidence would be beneficial [65]. Second, some of the studies relate to the same trial, which further restricts the number of primary studies available and limits the generalizability of our findings. Third, due to considerable heterogeneity across the interventions and outcome measures reported, it is difficult to identify which components of the patient portals, if any, influence the observed effects. Variations in portal design and functionalities also limit generalizability of the findings, and sustained engagement was a noted issue. Finally, outcomes related to safety and timeliness were underexplored and mostly subjectively reported. The lack of evidence on these important care domains further restricts the ability to fully determine whether patient portals meaningfully contribute to respiratory disease care.

### Implications for Research, Practice, and Policy

The findings of this review highlight the need for a stronger evidence base to support the implementation of patient portals in respiratory disease pathways. There is growing interest in the use of patient-facing digital health interventions, as evidenced by the recent National Institute for Health and Care Excellence guideline recommending several tools for COPD self-management (including 1 intervention included in this review) [41,66], and similar guidance for asthma is also underway [67]. However, the available research does not yet offer definite support for their routine use, highlighting the need for continued rigorous evaluation to support implementation efforts. In particular, the need for real-world evidence addressing digital engagement barriers is important. The recent NHS long-term plan may offer opportunities to integrate respiratory care–specific functions within national digital health platforms such as the NHS App [68], which can mitigate some of the access and engagement barriers we noted. For example, patients in England can already view test results, access care advice (including those specific to respiratory symptoms), and manage their medication list through the NHS App [68]. Therefore, facilitation efforts enabling wider use of national digital portals may offer some of the respiratory disease–specific benefits and allow coordinated management of multiple health conditions. However, as a precursor to this, further methodologically robust studies and adequately powered RCTs are crucial to address the limitations of the existing evidence base and to establish the validity of the reported benefits. This could include studies mapping specific functions to clinical outcomes and those with a longer follow-up period, including diverse groups such as adults and pediatric populations with asthma. Data collection approaches also need to be intersectional, recruiting a wide range of patient characteristics and across the various quality domains to identify user groups that would most benefit from these services.

### Conclusions

This comprehensive assessment of primary studies does not provide support for meaningful improvements in the quality of care for asthma and COPD using patient portals. The evidence on COPD is restricted to a few large observational trials. While studies report some disease-specific gains (eg, modest asthma control improvements and reduced hospitalizations in COPD), these findings are based on a small number of predominantly low-quality studies, which limits confidence in the overall evidence. This review strengthens the existing literature by offering a synthesis of the most up-to-date findings on the impacts of patient portals in asthma and COPD, addressing the fragmented focus of prior reviews that grouped various technologies together. However, the available research provides insufficient confirmation of meaningful care quality improvements, suggesting that reliance on patient portals for routine clinical management of asthma and COPD should be approached cautiously. If patient portals are to play a greater role in supporting self-management and quality of care, further studies on impacts and equity are required.

## Supplementary material

10.2196/83483 Multimedia Appendix 1 Full search strategy and additional study details.

10.2196/83483 Multimedia Appendix 2 Key characteristics of the included studies evaluating patient portals in adult asthma and chronic obstructive pulmonary disease (COPD). The table details the key characteristics of the 16 included papers, which include the study design, population included, intervention features, and comparator.

10.2196/83483 Checklist 1 PRISMA 2020 checklist.

10.2196/83483 Checklist 2 PRISMA abstract checklist.

10.2196/83483 Checklist 3 SWiM checklist.

## Abbreviations

ACQ: Asthma Control Questionnaire
COPD: chronic obstructive pulmonary disease
EHR: electronic health record
GRADE: Grading of Recommendations Assessment, Development, and Evaluation
IOM: Institute of Medicine
NHS: National Health Service
PRISMA: Preferred Reporting Items for Systematic Reviews and Meta-Analyses
PRISMA-S: Preferred Reporting Items for Systematic Reviews and Meta-Analyses literature search extension
PROSPERO: International Prospective Register of Systematic Reviews
QoL: quality of life
RCT: randomized controlled trial
SWiM: Synthesis Without Meta-analysis
