# Supplementary material for: Impact of Patient Portals on Asthma and Chronic Obstructive Pulmonary Disease–Related Quality-of-Care Outcomes: Systematic Review
Source: Online J Public Health Inform. 2026 Aug 24;18:e83483. doi: 10.2196/83483 (PMC13502935; doi:10.2196/83483)
Supplement: Supplementary file 1 [file ojphi-v18-e83483-s001.pdf]

Multimedia Appendix 1

A1. Full Search Strategy for all databases

Medline(R)

1. Asthma/
2. Chronic Obstructive Pulmonary Disease/
3. Asthma-Chronic Obstructive Pulmonary Disease Overlap Syndrome/
4. (asthma or COPD or "chronic obstructive pulmonary disease").mp.
5. 1 or 2 or 3 or 4
6. Patient Portals/
7. (patient portal* or family portal* or citizen portal* or consumer portal* or personalised portal* or patient platform* or consumer platform* or e-health or ehealth or m-health or mhealth or mobile app* or smartphone app*).mp.
8. (electronic medical record* or electronic health record*).mp.
9. 6 or 7 or 8
10. Medication Compliance/ or Medication Adherence/
11. symptom check*.mp.
12. Lung Function/ or lung function.mp.
13. Health Care Utilization/ or service-use.mp.
14. Hospitalization/ or (hospitali?ation adj2 rate*).mp.
15. Disease Exacerbation/ or exacerb*.mp.
16. Satisfaction/ or Patient Satisfaction/ or Life Satisfaction/
17. (quality of life or QoL).mp.
18. Health Care Quality/ or quality of care.mp.
19. Health Care Costs/ or Hospital Costs/ or Hospitalization Costs/
20. cost.mp.
21. cost-effectiveness.mp.
22. ((patient adj3 (safety or harm)) or misdiagnos*).mp.
23. 10 or 11 or 12 or 13 or 14 or 15 or 16 or 17 or 18 or 19 or 20 or 21 or 22
24. 5 and 9 and 23
25. limit 24 to (english language and ("all adult (19 plus years)" or "adolescent (13 to 18 years)" or "young adult (19 to 24 years)" or "adult (19 to 44 years)" or "young adult and adult (19-24 and 19-44)" or "middle age (45 to 64 years)" or "middle aged (45 plus years)" or "all aged (65 and over)" or "aged (80 and over)"))

Embase

1. exp Asthma/ or Chronic Obstructive Pulmonary Disease/
2. (asthma or COPD or "chronic obstructive pulmonary disease").mp.
3. Asthma-Chronic Obstructive Pulmonary Disease Overlap Syndrome/
4. 1 or 2 or 3
5. exp Patient Portals/
6. (patient portal or family portal or citizen portal or consumer portal or personalised portal or patient platform or consumer platform or e-health or m-health or mobile app* or smartphone app*).mp.
7. (electronic medical record* or electronic health record*).mp.
8. 5 or 6 or 7

9. medication compliance/ or medication adherence/
10. (symptom check* or lung function).mp. or lung function/ [mp=title, abstract, heading word, drug trade name, original title, device manufacturer, drug manufacturer, device trade name, keyword heading word, floating subheading word, candidate term word]
11. Hospitalization/ or (hospitali?ation adj2 rate*).mp. or disease exacerbation/ or exacerb*.mp. [mp=title, abstract, heading word, drug trade name, original title, device manufacturer, drug manufacturer, device trade name, keyword heading word, floating subheading word, candidate term word]
12. satisfaction/ or patient satisfaction/ or life satisfaction/
13. (quality of life or QoL).mp.
14. health care quality/ or quality of care.mp.
15. "health care cost"/ or cost.mp. or "hospital cost"/ or "hospitalization cost"/ [mp=title, abstract, heading word, drug trade name, original title, device manufacturer, drug manufacturer, device trade name, keyword heading word, floating subheading word, candidate term word]
16. cost-effectiveness.mp.
17. ((patient adj3 (safety or harm)) or misdiagnos*).mp. [mp=title, abstract, heading word, drug trade name, original title, device manufacturer, drug manufacturer, device trade name, keyword heading word, floating subheading word, candidate term word]
18. 9 or 10 or 11 or 12 or 13 or 14 or 15 or 16 or 17
19. 4 and 8 and 18
20. limit 19 to (english language and (adolescent <13 to 17 years> or adult <18 to 64 years> or aged <65+ years>))

PsycINFO

1. Asthma/
2. Chronic Obstructive Pulmonary Disease/
3. (ACOS or ACO).mp.
4. (asthma or COPD or "chronic obstructive pulmonary disease").mp.
5. 1 or 2 or 3 or 4
6. Patient Portals/
7. Electronic Medical Records/
8. Mobile Applications/
9. (patient portal* or family portal* or citizen portal* or consumer portal* or personalised portal* or patient platform* or consumer platform* or e-health or ehealth or m-health or mhealth or mobile app* or smartphone app*).mp.
10. (electronic medical record* or electronic health record*).mp.
11. 6 or 7 or 8 or 9 or 10
12. Medication Compliance/ or Treatment Compliance/
13. Symptom Assessment/ or symptom check*.mp.
14. Lung Function.mp.
15. Health Care Utilization/
16. Hospitalization/
17. (hospitali?ation adj2 rate*).mp.
18. (exacerbat* or disease exacerbation).mp.
19. Patient Satisfaction/
20. Life Satisfaction/
21. Quality of Life/

22. (quality of life or QoL).mp.
23. Health Care Quality/ or quality of care.mp.
24. Health Care Costs/ or Medical Costs/
25. cost.mp.
26. cost-effectiveness.mp.
27. ((patient adj3 (safety or harm)) or misdiagnos*).mp.
28. 12 or 13 or 14 or 15 or 16 or 17 or 18 or 19 or 20 or 21 or 22 or 23 or 24 or 25 or 26 or 27
29. 5 and 11 and 28
30. limit 29 to (english language and ("200 adolescence (age 13 to 17 yrs)" or "300 adulthood (age 18 yrs and older)" or "320 young adulthood (age 18 to 29 yrs)" or "340 thirties (age 30 to 39 yrs)" or "360 middle age (age 40 to 64 yrs)" or "380 aged (age 65 yrs and older)" or "390 very old (age 85 yrs and older)"))

CINAHL
S1: (MH "Asthma") OR (MH "Pulmonary Disease, Chronic Obstructive") OR (MH "Asthma, Exercise-Induced") OR (asthma OR COPD OR "chronic obstructive pulmonary disease")

S2: (MH "Patient Portals") OR (MH "Medical Records, Electronic") OR (MH "Mobile Applications") OR ((MH "Telemedicine") OR (MH "Consumer Health Informatics")) OR ((patient portal* OR family portal* OR citizen portal* OR consumer portal* OR personalised portal* OR patient platform* OR consumer platform* OR e-health OR ehealth OR m-health OR mhealth OR mobile app* OR smartphone app*).TX.) OR ((electronic medical record* OR electronic health record*).TX.)

S3: (MH "Medication Compliance") OR (MH "Symptom Assessment") OR (MH "Lung Function Tests") OR (MH "Health Services Utilization") OR (hospitali?ation N2 rate*).TX. OR symptom check*.TX. OR exacerb*.TX. OR (MH "Patient Satisfaction") OR (MH "Life Satisfaction") OR (MH "Quality of Life") OR (MH "Health Care Quality") OR ("quality of life" OR QoL)

S4: quality of care.TX. OR (MH "Health Care Costs") OR cost.TX. OR cost-effectiveness.TX. OR (((patient N3 (safety OR harm)) OR misdiagnos*).TX**)**

Subquery S5:
S3 OR S4: ((MH "Medication Compliance") OR (MH "Symptom Assessment") OR (MH "Lung Function Tests") OR (MH "Health Services Utilization") OR (hospitali?ation N2 rate*).TX. OR symptom check*.TX. OR exacerb*.TX. OR (MH "Patient Satisfaction") OR (MH "Life Satisfaction") OR (MH "Quality of Life") OR (MH "Health Care Quality") OR ("quality of life" OR QoL)) OR (quality of care.TX. OR (MH "Health Care Costs") OR cost.TX. OR cost-effectiveness.TX. OR (((patient N3 (safety OR harm)) OR misdiagnos*).TX))

S1 AND S2 AND S5

Google Scholar

URL: https://scholar.google.com/

Date searched: 5th January 2025 and 11th March 2026

Search string:
(“asthma” OR “COPD” OR “chronic obstructive pulmonary disease”) AND (“patient portal” OR “online portal”)

Filters:

- Sorted by relevance
- No date restriction

Screening approach:

- First 200 results screened in order of relevance

A2: Additional study details and socio-demographic indicators reported.

AUTHOR, DATE (COUNTRY)	AIMS/OBJECTIVE (Transcribed verbatim)	AUTHOR'S CONCLUSIONS	POPULATION DEMOGRAPHY	
			Ethnicity	Other
Acharya and Sarraf (2017) [29] (USA)	"...to study existing asthma applications and propose a holistic approach to asthma care and management by developing a novel Android application that provides a real-time, multi-way communication between patients and providers (healthcare, insurance, food, drug, etc.)."	The developed application eliminates manual methods of communication with a real-time proactive and automated approach to improve the quality and cost of care. The majority of the participants agreed that real-time feedback from healthcare providers helped them to better manage their condition. Although we believe that the closed loop communication between providers and patients can reduce the number of office visits and reduce cost, just 50% of the patients agreed, and the rest still prefer faceto-face communication with their medical doctors. Furthermore, almost all of the healthcare providers prefer to use the new technology to communicate with their patients, and a large number of the healthcare providers reported that the use of pro-care application reduced the number of office visits	Not stated	Not stated

AUTHOR, DATE (COUNTRY)	AIMS/OBJECTIVE (Transcribed verbatim)	AUTHOR'S CONCLUSIONS	POPULATION DEMOGRAPHY	
			Ethnicity	Other
		for their patients during the evaluation period.		
Ahmed et al (2016) [30] (Canada)	"...to compare the impact of access to a Web-based asthma self-management patient portal linked to a case-management system (MAP) over 6 months compared with usual care on asthma control and quality of life."	The MAP intervention had a significant impact on improving asthma-related quality of life and other related outcomes including self-efficacy, depressive symptoms, and beliefs about medication. Furthermore, the effects of MAP were maintained at 6 months and began to decrease once the use of MAP ended between 6 and 9 months, suggesting that ongoing support might result in reduced exacerbations in the long term as opposed to usual care alone.	Not stated	**Education,** n (%)<br>Higher school**=** Intervention: 32 (68%), Control: 26 (51%)<br>High school equivalent**=** Intervention: 13 (28%), Control: 22 (43%)<br><High school**=** Intervention: 1 (2%), Control: 1 (2%)<br>Missing**=** Intervention: 1 (2%), Control: 2 (4%)

AUTHOR, DATE (COUNTRY)	AIMS/OBJECTIVE (Transcribed verbatim)	AUTHOR'S CONCLUSIONS	POPULATION DEMOGRAPHY	
			Ethnicity	Other
Alladina et al (2024) [31] (USA)	"…to evaluate usage, usability, and satisfaction with the platform; patient reported asthma experiences; and ease of implementation of the app."	Most patients and HCPs were satisfied with Amaze. The Amaze platform may help patients and healthcare professionals monitor asthma status, which could improve asthma control.	n (%)<br>American Indian or Alaska Native= 1 (0.8%)<br>Asian= 4 (3.3%)<br>Black or African American= 11 (9.2%)<br>Multiple= 5 (4.2%)<br>White= 94 (78.3%)<br>Other= 3 (2.5%)<br>Missing= 2 (1.7%)	**Employment,** n (%)<br>Disabled= 5 (4.2%)<br>Employed= 55 (45%)<br>Homemaker= 9 (7.5V%)<br>Retired=11 (9.2%)<br>Student=30 (25.0%)<br>Unemployed=5 (4.2%)<br>Other= 3 (2.5%)<br>Missing= 3 (2.5%)<br><br>**Education**, n (%)<br>Secondary/high school or lower= 34 (28.3%)<br>Associate degree, technical or trade school=8 (6.7%)<br>College/university degree=47 (39.2%)<br>Postgraduate school=26 (21.7%)<br>Other= 1(0.8%)<br><br>**Household total income over the past 12 months**, n (%)<br>Less than $75,000= 28 (23.3%)<br>$75,000 or more= 66 (55.0%)<br>Missing= 3 (2.5%)<br>Prefer not to answer= 23 (19.2%)

AUTHOR, DATE (COUNTRY)	AIMS/OBJECTIVE (Transcribed verbatim)	AUTHOR'S CONCLUSIONS	POPULATION DEMOGRAPHY	
			Ethnicity	Other
Apter et al (2019) [32] (USA)	"To estimate the effect on asthma outcomes of an intervention using home visits by community health workers plus training in patient portals compared to usual care and portal training only."	For low-income adults with uncontrolled asthma, portal access and community health workers produced small incremental benefits. Home visits with emphasis on self management education might be necessary to facilitate patient-clinician communication and to improve outcomes for asthma.	n (%)<br>**Non-Hispanic Black/African-American=**Intervention: 116 (76.8%), Control: 111 (74.0%)<br><br>**Hispanic or Latino=** Intervention: 31 (20.5%),Control: 35 (23.3%)<br><br>**Non-Hispanic White=** Intervention: 1 (0.7%),Control: 3 (2.0%)<br><br>**Other=**Intervention: 3 (2.0%),Control: 1 (0.7%)	n(%)<br>Household Income:<br>• Less than $30,000= Intervention: 119 (79%), Control: 115 (77%)<br>• $30,000 - $49,999= Intervention: 12 (7.9%), Control: 7 (4.7%)<br>• $50,000 - $99,999= Intervention: 7 (4.6%), Control: 4 (2.7%)<br>• $100,000 or over= Intervention: 0 (0%), Control: 2 (1.3%)<br>• Decline to answer= Intervention: 13 (8.6%), Control: 22 (14.7%)<br><br>Insurance Type:<br>• Medicaid: Intervention= 90 (59.6%), Control: 100 (66.7%)<br>• Medicare only: Intervention= 21 (13.9%), Control: 8 (5.3%)<br>• Self-pay: Intervention= 2 (1.3%), Control: 2 (1.3%)<br>• Commercial with or without Medicare= Intervention: 38 (25.2%), Control: 40 (26.7%)<br><br>Education Attainment

AUTHOR, DATE (COUNTRY)	AIMS/OBJECTIVE (Transcribed verbatim)	AUTHOR'S CONCLUSIONS	POPULATION DEMOGRAPHY	
			Ethnicity	Other
				(Highest Level Achieved):<br>• 8th grade or less= Intervention: 12 (7.9%), Control: 10 (6.7%)<br>• Some high school= Intervention: 38 (25.2%), Control: 29 (19.3%)<br>• High school graduate or G.E.D.= Intervention: 50 (33.1%), Control: 61 (40.7%)<br>• Some college or trade school= Intervention: 34 (22.5%), Control: 32 (21.3%)<br>• College graduate: Intervention= 17 (11.3%), Control: 18 (12.0%)<br><br>Literacy (S-TOFHLA):<br>• Mean score: Intervention= 25 ± 14, Control: 25 ± 13

AUTHOR, DATE (COUNTRY)	AIMS/OBJECTIVE (Transcribed verbatim)	AUTHOR'S CONCLUSIONS	POPULATION DEMOGRAPHY	
			Ethnicity	Other
Apter et al (2020) [33] (USA)	"To describe portal activities and association with 12-month outcomes among low-income patients with asthma formally trained in portal use."	Patients with uncontrolled asthma used the portal irregularly if at all, despite in-person training. Usage was not associated with regular appointments or with clinical outcomes. Patient portals need modification to accommodate low income patients with uncontrolled asthma.	Not stated	Not stated
Cruz-Correia et al (2007) [34] (Portugal)	"This paper aims at describing and evaluating the use of P'ASMA – a web based asthma monitoring tool, namely the opinion of users (patients) and adherence to monitoring in comparison to standard paper based tools."	Use of a mobile engagement platform, such as POPET, can have a significant impact on health outcomes and quality of life in both AR and asthma, potentially decreasing the number of hospital admissions, repeat doctor visits, and losses in productivity. Improvements were seen in domains related to activity, productivity, perception of disease, and emotion.	Not stated	Education= Median 11 years of schooling (range 4 to 18)

AUTHOR, DATE (COUNTRY)	AIMS/OBJECTIVE (Transcribed verbatim)	AUTHOR'S CONCLUSIONS	POPULATION DEMOGRAPHY	
			Ethnicity	Other
Localio et al (2022) [35] (USA)	"To assess electronic health record patient portal use among Spanish-speaking patients with asthma compared to English-speaking patients and identify barriers to use."	English-only patient portals may not meet the needs of Spanish-speaking patients with uncontrolled asthma. Health systems serving Spanish-speaking communities should implement patient portals in Spanish.	Hispanic/Latino n(%)= 52 (17%) overall	n(%)<br>Education (High school graduate or higher):<br>Overall= 212 (70%)<br>English speakers= 190 (76%)<br>Spanish speakers= 22 (42%)<br><br>Insurance:<br>Medicaid=190 (63%)<br>Spanish speakers=42 (81%)<br>Intervention/Control: Not stated<br><br>Health Literacy (S-TOFHLA score) Median (range):<br>Overall = 31 (0–36)<br>English = 32 (0–36)<br>Spanish = 22 (0–36)
Mammen et al (2021) [36] (USA)	"We describe the process of contextually grounded intervention development, the resulting technology, and proof-of-concept testing in a small, real-world sample of adult patients in a large urban medical centre."	Smartphone telemedicine may be an effective means to improve outcomes and deliver asthma care remotely.	n(%)<br>African American= 4 (57%)<br>Hispanic/Latino= 2 (29%)<br>Caucasian= 1 (14%)	Yes, n (%)<br>Low income i.e.,<200% US Federal poverty level= 5 (71%)<br>Employed full time =5 (71%)<br>Public insurance= 4 (57%)<br>High school graduate= 5 (71%)

AUTHOR, DATE (COUNTRY)	AIMS/OBJECTIVE (Transcribed verbatim)	AUTHOR'S CONCLUSIONS	POPULATION DEMOGRAPHY	
			Ethnicity	Other
Mammen et al (2022) [37] (USA)	"We describe the process of contextually grounded intervention development, the resulting technology, and proof-of-concept testing in a small, real-world sample of adult patients in a large urban medical centre."	We conclude that smartphone telemedicine could substantially improve clinical asthma management, adherence to guidelines, and patient outcomes.	*n(%)*<br>*Patients:*<br>Black=15 (50.0%)<br>White= 6 (20.0%)<br>Hispanic/Latino= 4 (13.3%)<br>Multiracial =4 (13.3%)<br>Asian=1 (3.3%)<br><br>Providers:<br>White=3 (75%)<br>Asian=1 (25%)	n(%)<br>Patients:<br>Insurance (public)=23 (76.7%)<br>Education (Highschool or less) =16 (53.3%)<br>Marital status (Single) =19 (66.3%)<br>Transportation (public, no car) =16 (53.3%)<br>Employed full or part time= 19 (63.4%)<br>Average household Income (in $1,000 USD) =27.53 (SD 13.97)
Martínez Nicolás et al (2019) [38] (Spain)	"...to determine the extent to which an EPP was associated with improvements in health service use."	We conclude that smartphone telemedicine could substantially improve clinical asthma management, adherence to guidelines, and patient outcomes.	80% Spanish born	n/a
Morita et al (2019) [39] (Canada)	" The aim of this study was to design, develop, and assess the utilization of an asthma collaborative self-management (CSM) platform (breathe) using content based on international evidence-based clinical guidelines."	Individuals with asthma reported good usability and high satisfaction levels, reacted to breathe notifications, and had confidence in the platform's assessment of asthma control. Strong utilisation was seen at the intervention's initiation, followed by a rapid reduction in use. Patient reminders, physician visits, and being aged 50 years and above	Caucasian %=97.1%	Not stated

AUTHOR, DATE (COUNTRY)	AIMS/OBJECTIVE (Transcribed verbatim)	AUTHOR'S CONCLUSIONS	POPULATION DEMOGRAPHY	
			Ethnicity	Other
		were associated with higher utilisation.		
Rudin et al (2019) [40] (USA)	"...to evaluate the feasibility of between-visit asthma symptom monitoring via our mHealth intervention by conducting a mixed-methods analysis of our implementation."	Implementation of a simple, clinically integrated mHealth app and practice model was feasible and resulted in a high and sustained rate of weekly questionnaire completion and minimal burden on clinical staff.	Patients only, n(%)<br>White= 20 (77%)<br>Non white= 6 (23%)	Education, patients only, n(%)<br>Bachelors= 14 (54%)<br>Non-bachelors= 9 (35%)<br>Missing= 3 (12%)
Taylor et al (2023) [41] (UK)	"...to determine the acceptability, feasibility, and utility of deploying the co-developed COPD digital service alongside routine clinical care for people with severe COPD."	Implementation of a simple, clinically integrated mHealth app and practice model was feasible and resulted in a high and sustained rate of weekly questionnaire completion and minimal burden on clinical staff.	Not stated	58% of the intervention and 63% of the control cohort were resident in postcodes in the most socioeconomically deprived quintiles.

AUTHOR, DATE (COUNTRY)	AIMS/OBJECTIVE (Transcribed verbatim)	AUTHOR'S CONCLUSIONS	POPULATION DEMOGRAPHY	
			Ethnicity	Other
To et al (2020) [42] (Canada)	"...to determine the acceptability, feasibility, and utility of deploying the co-developed COPD digital service alongside routine clinical care for people with severe COPD."	Use of the Breathe app is not associated with changes in health services use in adults with asthma.	*Ethnic concentration* (% lowest and highest quintiles)<br><br>Breathe vs.non-breathe<br>1= 31.3% vs.28.7%;<br>5(highest)= 7.2% vs. 7.3%<br><br>Breathe vs. population control<br>1= 26.5% vs. 32.3%;<br>5(highest)=7.6% vs. 6.1%	*Urban residence %*<br>Breathe vs. non-breathe=87.9% vs. 88.7%<br>Breathe vs.population control=87.9% vs. 84.8%<br><br>*Income*(% lowest and highest quintiles)<br>*Breathe vs.non-breathe*<br>*1= 18.6% vs. 18.1%;*<br>*5(highest)= 21.3% vs. 20.8%*<br>*Breathe vs. population control*<br>*1= 12.1% vs. 12.1%;*<br>*5(highest)=18.2% vs. 17.9%*<br><br>*Deprivation* (% lowest and highest quintiles)<br>Breathe vs.non-breathe<br>1= 24.7% vs. 24.5%;<br>5(highest)= 22.1% vs. 21.9%<br><br>Breathe vs. population control<br>1= 30.3% vs. 30.1%;<br>5(highest)=16.7% vs. 16.2%<br><br>*Residential instability* (% lowest and highest quintiles)<br>Breathe vs.non-breathe<br>1= 8.4% vs. 9.0%;<br>5(highest)= 24.3% vs. 25.0%

AUTHOR, DATE (COUNTRY)	AIMS/OBJECTIVE (Transcribed verbatim)	AUTHOR'S CONCLUSIONS	POPULATION DEMOGRAPHY	
			Ethnicity	Other
				Breathe vs. population control<br>1= 9.8% vs. 10.6%;<br>5(highest)=22.7% vs. 21.2%

AUTHOR, DATE (COUNTRY)	AIMS/OBJECTIVE (Transcribed verbatim)	AUTHOR'S CONCLUSIONS	POPULATION DEMOGRAPHY	
			Ethnicity	Other
Tossaint-Schoenmakers et al (2021) [43] (Netherlands)	"...to assess the effect of patient characteristics (gender, age, education, and chronic disease) on the self-efficacy and perceived usability of an online patient portal that communicates diagnostic test results."	This study showed that the higher-educated users of a patient portal scored lower on usability and self-efficacy. Usability was also lower for older people and for patients with asthma or chronic obstructive pulmonary disease. The results portal is not tailored for different groups. Further research should investigate which factors from a patient's perspective are essential to tailor the portal for different groups and how a result portal can be optimally integrated within the daily practice of a doctor.	Not stated	Education, n(%)<br>Low (no education, high school)=96 (12.8%)<br>Intermediate (intermediate vocational education)=220 (29.4%)<br>High (bachelor's degree, master's degree, doctorate)=423 (56.6%)
Zijp et al (2024) [44] (Netherlands)	"...to assess the feasibility, acceptability, usability and safety of Astmakompas from patient and healthcare professioanls perspectives, and secondarily to explore potential effects on asthma control, medication use, Quality of	Astmakompas shows promise for further implementation and research. Future randomized studies with longer evaluation periods are crucial to assess the tool's impact on asthma outcomes and workload. It is important that HCPs guide and tailor interventions to meet the patient's individual needs and circumstances.	Not stated	Education (*patients only*) n(%)<br><br>Primary education= 0 (0.0%)<br>Lower vocational education= 0 (0.0%)<br>Intermediate general secondary education= 0 (0.0%)<br>Senior secondary vocational education= 5 (35.7%)<br>Senior general secondary education=1 (7.1%)

AUTHOR, DATE (COUNTRY)	AIMS/OBJECTIVE (Transcribed verbatim)	AUTHOR'S CONCLUSIONS	POPULATION DEMOGRAPHY	
			Ethnicity	Other
	Life, self-efficacy and (in)direct costs."			Higher professional education= 6 (42.9%)<br>University education= 2 (14.3%)
