# Supplementary material for: Impact of Patient Portals on Asthma and Chronic Obstructive Pulmonary Disease–Related Quality-of-Care Outcomes: Systematic Review
Source: Online J Public Health Inform. 2026 Aug 24;18:e83483. doi: 10.2196/83483 (PMC13502935; doi:10.2196/83483)
Supplement: Supplementary file 2 [file ojphi-v18-e83483-s002.pdf]

Multimedia Appendix 2

Table S1. Key characteristics of the included studies evaluating the impact of patient portals in adult asthma and chronic obstructive pulmonary disease (COPD).

Author, date (country)	Study details<br>a. Design<br>b. Setting<br>c. Data collection period	Condition type	Exclusion criteria	Number of participants included	Population demography (age, sex)*	DHI name/ function	Comparator
Acharya and Sarraf (2017), [29] (United States)	a. Pre-post quasi experimental<br><br>b. Tertiary care (regional trauma centre and partner clinical practices)<br><br>c. 12 months-quarterly collection	ASTHMA	Not stated	N= 116 (Healthcare providers= 36 Patients=68 IT personnel= 12)	Patients only<br>**Age range**: 18-44 years<br><br>**Sex** (n)<br>*Females*: 23, *Males*: 45 (Described as 1:2 ratio of female: male)	Pro-care framework<br><br>• Journal entry<br>• Asthma diary<br>• Peak flow chart<br>• Notification<br>• Action plan<br>• Inhaler log<br>• Call 911	Not stated
Ahmed et al (2026) [30] (Canada)	a. Pilot RCT<br><br>b. Tertiary care(pulmonary clinics located in 2 tertiary hospitals)<br><br>c.3, 6 and 9 months	ASTHMA	COPD, serious medical conditions (e.g. lung cancer) or severe mobility restrictions)	N= 100 (Intervention=49 Control= 51)	**Age** (n, %)<br>*0-39 years*= Intervention: (20, 43%), Control: (14,27%)<br>*40-49 years*= Intervention: (10, 21%), Control: (13, 25%)<br>*50-59 years*= Intervention: (12, 26%), Control: (15, 29%)<br>*>60 years*= Intervention: (5, 11%), Control: (9,18%)<br><br>**Sex** (n, %)<br>*Females*: Intervention: 32 (68%), Control: 33 (65%)	My Asthma Portal (MAP)<br><br>• Personal health information<br>• Access educational resources<br>• Monitor and receive feedback on self-management (color-coding, email alerts)<br>• Communication with nurse case manager<br><br>• Provider function with communication log and alerts regarding new participants, action plan	Usual care (Written action plan, regular pulmonologist care, education and follow up from asthma nurse but not MAP access)

Author, date (country)	Study details<br>a. Design<br>b. Setting<br>c. Data collection period	Condition type	Exclusion criteria	Number of participants included	Population demography (age, sex)*	DHI name/ function	Comparator
						issues, or medication changes	
Alladina et al (2024) [31] (United States)	a. Observational study<br><br>b. Multi-centre (a community allergy practice and a pulmonology practice at an academic hospital)<br><br>c. 7 months	ASTHMA	Diagnosis of chronic obstructive pulmonary disease or other non-asthma pulmonary disease and those without a smartphone with internet access (operating system iOS 13 or newer/iPhone 8 or newer or Android operating system 8 or newer)	N= 157(patients= 152, healthcare providers= 5)	Patients only<br><br>**Age (**Mean): 38.7 years, SD=16.4<br><br>**Sex** (n, %)<br>*Females:* 93(78%)	Amaze™ digital platform<br><br>• Symptom log and trends<br>• Trigger log<br>• Medication use tracker<br>• Emergency visit log<br>• Patient-provider communication<br>• Air quality updates<br>• Appointment booking and view<br>• Personalised asthma plans<br>• Educational resources<br>• Provider facing dashboard for asthma control review and guidance	Not stated

Author, date (country)	Study details<br>a. Design<br>b. Setting<br>c. Data collection period	Condition type	Exclusion criteria	Number of participants included	Population demography (age, sex)*	DHI name/ function	Comparator
Apter et al (2019) [32] (United States)	a. RCT<br><br>b. Multi-centre (outpatient family medicine practices, 2 general internal medicine, 2 pulmonary medicine, 1 allergy-immunology of academic medical center, and 1 primary care practice serving mainly Spanish-speaking patients)<br><br>c. 12 months	ASTHMA	Participants with severe psychiatric or cognitive problems or had used the portal more than 3 times	N= 301 (Half the participants randomized to receive four community health worker home visits in the first six months, each 20-30mins long)	**Age:** 18 years and older<br><br>**Sex** (n, %)<br>*Females*: Intervention= 136 (90.1%), Control= 134 (89.3%)	Epic MyChart portal<br><br>Patients were trained on:<br>• Reviewing upcoming appointments<br>• Scheduling appointments<br>• Reviewing medication lists<br>• Locating laboratory results<br>• Locating immunization records<br>• Requesting prescription refills<br>• Messaging<br>(Full list of portal functions not described)	Usual care plus portal training/ access without home visits

Author, date (country)	Study details<br>a. Design<br>b. Setting<br>c. Data collection period	Condition type	Exclusion criteria	Number of participants included	Population demography (age, sex)*	DHI name/ function	Comparator
Apter et al (2020) [33] (United States)	a. Secondary observational study<br><br>b. Multi-centre (4 primary care, 3 asthma speciality clinics and 1 service for mostly Latino/Hispanic patients)<br><br>c. 12 months	ASTHMA	Participants with severe psychiatric or cognitive problems or had used the portal more than 3 times	N= 301	**Age**: 18 years and older<br><br>**Sex**: Not stated	Epic MyChart portal<br><br>Patients were trained on:<br>• Reviewing upcoming appointments<br>• Scheduling appointments<br>• Reviewing medication lists<br>• Locating laboratory results<br>• Locating immunization records<br>• Requesting prescription refills<br>• Messaging<br>(Full list of portal functions not described)	Usual care plus portal training/ access without home visits

Author, date (country)	Study details<br>a. Design<br>b. Setting<br>c. Data collection period	Condition type	Exclusion criteria	Number of participants included	Population demography (age, sex)*	DHI name/ function	Comparator
Cruz-Correia et al (2007) [34] (Portugal)	a. RCT, Crossover trial<br><br>b. Tertiary care (Immuno-allergology outpatient clinic of a hospital)<br><br>c. 8 weeks	ASTHMA	Severe psychiatric, neurological, oncologic or immunologic disease , unable to use the Internet during the study period	N= 21	**Age** Mean ± SD (range): 29±10 years (18 to 62 years)<br><br>**Sex** n(%)<br>*Females*= 5 (71%), *Males*= 6 (29 %)	Portal for Assessment & Self-Management of Asthma (P'ASMA)<br><br>• Symptom and exacerbation logs<br>• Lung function log (PEF, FEV_1)<br>• Feedback<br>• Trend charts and asthma-control score<br>• Education library<br>• Messaging<br>• Automated e-mail alerts<br><br>For clinicians:<br>• Longitudinal monitoring and GINA control data view<br>• Customisable monitoring schedule (which variables, how often)	Paper asthma diary and action plan

Author, date (country)	Study details<br>a. Design<br>b. Setting<br>c. Data collection period	Condition type	Exclusion criteria	Number of participants included	Population demography (age, sex)*	DHI name/ function	Comparator
Localio et al (2022) [35] (United States)	a. Secondary observational study<br><br>b. Multi-centre(2 family medicine, 2 general internal medicine, 2 pulmonary, and 1 allergy-immunology outpatient practices)<br><br>c. 12 months	ASTHMA	Participants with severe psychiatric or cognitive problems or had used the portal more than 3 times	N= 301 (Half the participants randomized to receive four community health worker home visits, each 20-30mins long)	**Age** Median (range):Overall= 51 years (18-87), English= 50 years (18–84), Spanish= 53 years (24–87)<br><br>**Sex** n(%)<br>*Females*: Overall= 270 (90%), English= 223 (90%), Spanish= 47 (90%)	Epic MyChart portal<br><br>Patients were trained on:<br>• Reviewing upcoming appointments<br>• Scheduling appointments<br>• Reviewing medication lists<br>• Locating laboratory results<br>• Locating immunization records<br>• Requesting prescription refills<br>• Messaging<br>(Full list of portal functions not described)	English speaking vs. Spanish speaking users
Mammen et al (2021) [36] (United States)	a. Pilot (proof-of concept) study, Mixed methods<br><br>b. Primary Care<br><br>c. 3 months	ASTHMA	Age restricted to 18-40 years and patients with confounding comorbidities (e.g. heart failure chronic obstructive pulmonary disease, cystic fibrosis), pregnant women	N= 7	**Age** Mean (SD): 29.5 years (5.22)<br><br>**Sex** n (%)<br>*Females*(%): 6 (86%)	Technology Enabled Asthma Management System (TEAMS)<br><br>• Remote symptom monitoring<br>• Smartphone telemedicine visits<br>• Nurse-led self-management training<br>• EMR based clinical decision support software	Not stated

Author, date (country)	Study details<br>a. Design<br>b. Setting<br>c. Data collection period	Condition type	Exclusion criteria	Number of participants included	Population demography (age, sex)*	DHI name/ function	Comparator
Mammen et al (2022) [37] (United States)	a. Cohort study, Mixed methods<br><br>b. Primary Care<br><br>c. 6 months	ASTHMA	Without smartphone ownership, disease comorbidities and age range restricted to 18-44 years and others excluded.	N= 35 (Patients= 30, healthcare providers= 5)	**Age** Mean (SD): Patients= 32.97 years (6.33), Providers=36.50 years (15.33)<br><br>**Sex** n (%)<br>*Females*: Patients= 19 (63.3%), Providers= 4 (100%)	Technology Enabled Asthma Management System (TEAMS)<br><br>• Remote symptom monitoring<br>• Smartphone telemedicine visits<br>• Nurse-led self-management training<br>• EMR based clinical decision support software	Not stated
Martínez Nicolás et al (2019) [38] (Spain)	a. Quasi-experimental interrupted time-series<br><br>secondary Care<br><br>c. 4 years 8 months (April 2012-December 2016)	COPD	Patients who did not receive treatment at the study sites	N= 909,724	**Age** Mean: 42 years<br><br>**Sex** (%)<br>*Females*= 55%	An Electronic Patient Portal (EPP)<br><br>• View/ modify their patient profiles<br>• Schedule / manage appointments<br>• Medical record view<br>• Message healthcare staff<br>• Videoconference with medical staff<br>• Upload personal diagnostics	Not stated

Author, date (country)	Study details<br>a. Design<br>b. Setting<br>c. Data collection period	Condition type	Exclusion criteria	Number of participants included	Population demography (age, sex)*	DHI name/ function	Comparator
Morita et al (2019) [39] (Canada)	a. Secondary observational study<br><br>b. Multi-centre(6 primary care and 2 asthma specialty clinics)<br><br>c. 12 months	ASTHMA (well- to somewhat-well-controlled)	Diagnosis of other chronic lung disease (e.g. cystic fibrosis, chronic obstructive lung disease or bronchiectasis), without familiarity with and access to a mobile smartphone, laptop and desktop computer	N= 138	**Age** Mean (SD)= 45.3years (15.8)<br><br>**Sex** (%)<br>*Females*= 72.5%	Breathe<br><br>• Risk-reduction messaging<br>• Display of environmental conditions<br>• Daily and weekly symptom assessment<br>• Real-time access to personalised asthma action plan and medication use<br>• Reminders and alerts<br>• Historical data review<br>• Disease journal	Not stated
Rudin, et al (2019) [40] (United States)	a. Feasibility trial, Mixed-methods<br><br>b. Ambulatory Care<br><br>c. 25 weeks	ASTHMA	Cognitive impairment, non-regular use of a smart phone	N= 32 (Patients= 26, Health care professionals=6)	Patients only<br>**Age** Mean (SD)= 54 years (16)<br><br>**Sex** N(%)<br>*Females*= 19 (73%), *Males*= 7 (27%)	An mhealth App and Practice Model<br><br>• Symptom check and notifications<br>• Patient's review of symptoms on app<br>• Clinician's review of symptoms within the EHR	Not stated

Author, date (country)	Study details<br>a. Design<br>b. Setting<br>c. Data collection period	Condition type	Exclusion criteria	Number of participants included	Population demography (age, sex)*	DHI name/ function	Comparator
Taylor, et al (2023) [41] (United Kingdom)	a. Prospective observational cohort study<br><br>b. Secondary Care<br><br>c. 1 year 11 months (September 2019 - August 2021)	COPD	Lack of capacity for consent and communication barrier, without daily access to a smartphone, tablet or computer with internet access	N= 498 (Intervention= 83, control= 415)	**Age** Mean(SD): Intervention= 64.4 years (9.3), Control= 64.6 years (9.1)<br><br>**Sex** (%)<br>*Females*: Intervention= 63.9%, Control= 63.%	Lenus COPD support service<br><br>• Patient application to enter Patient Reported Outcomes (PRO) with further self-management support website<br>• Clinician dashboard where structured data from EHRs, PROs, wearables, and home non-invasive ventilation machines are displayed<br>• Asynchronous messaging	Non-user of the intervention
To et al (2020) [42] (Canada)	a. Quasi-experimental difference-in-difference<br><br>b. Primary Care<br><br>c. 3 years	ASTHMA	Diagnosis of other chronic lung disease (e.g. cystic fibrosis, chronic obstructive lung disease or bronchiectasis), without familiarity with and access to a mobile smartphone, laptop and desktop computer	N= 677 (Intervention= 132, internal control= 149, external control= 396)	**Age** Mean (SD): Breathe vs. non-breathe= 46.5 years (15.74) vs. 46.5 years (15.65),<br>Breathe vs. population control= 44.9 years (15.72) vs. 44.9 years (15.72)<br><br>**Sex (%)**<br>*Females:* Breathe vs. non-breathe= 71.0% vs. 71.87%, Breathe vs. population control= 72.7% vs. 72.7%	Breathe<br><br>• Risk-reduction messaging<br>• Display of environmental conditions<br>• Daily and weekly symptom assessment<br>• Real-time access to personalised asthma action plan<br>• Reminders and alerts<br>• Historical data review<br>• Disease journal	Receiving "equivalent quality" of asthma care

Author, date (country)	Study details<br>a. Design<br>b. Setting<br>c. Data collection period	Condition type	Exclusion criteria	Number of participants included	Population demography (age, sex)*	DHI name/ function	Comparator
Tossaint-Schoenmakers et al (2021) [43] (Netherlands)	a. Cross-sectional<br><br>b. Community setting<br><br>c. 7 months (December 2019 - July 2020)	ASTHMA OR COPD	Not stated	N= 748	**Age** Mean (SD): 52.8years (16.4)<br><br>**Sex** n (%)<br>*Females*: 428 (57.2%), *Males*: 314 (42.0%)	Web-based portal<br><br>• Blood-test results<br>• Information regarding the test, outcomes and action plan<br>• Visual support using traffic light colours to indicate whether the outcome is within the normal range	Not stated
Zijp, et al (2024) [44] (Netherlands)	a. Pre-post quasi experimental, Mixed methods<br><br>b. Secondary Care<br><br>c. 12 weeks	ASTHMA (Uncontrolled)	Respiratory disease other than asthma or a non-reversible airway obstruction and not having access to smartphone or internet	N=18 (Patients= 14, providers= 4)	**Age** Mean (range): Patients= 47.6 years (21–71), Providers= 44 years (32–59)<br><br>**Sex** n (%)<br>*Females*: Patients= 10 (71.4%), Providers= 4 (100%)	Astmakompas<br><br>• Asthma monitoring for patients using an app with wireless spirometer<br>• Patient-provider communication<br>• HCP facing portal to view patient data and monitor asthma control<br>• Disease alerts for healthcare professionals<br>• Digital action plan and guidance on asthma management	Not stated

Table details the key characteristics of the 16 included papers on patient portal use in asthma and COPD. Details include the author name, publication year and country, study design (randomised controlled trials, observational, interrupted time series etc.), study population, sample size, intervention features and comparator. Where, EHR= Electronic Health Records; IT= Information Technology; MAP= My Asthma Portal; P'ASMA= Portal for Assessment & Self-Management of Asthma; POPET= Physician On call Patient Engagement Trial; PRO= Patient Reported Outcomes; RCT= Randomised Controlled Trials; TEAMS= Technology Enabled Asthma Management System. *Additional population demography indicators where available is presented as Supplementary File, Table 2. This table also includes the aims/objectives of the included studies and the authors' conclusions.
